# A Markovian Approach towards Bacterial Size Control and Homeostasis in Anomalous Growth Processes

**DOI:** 10.1038/s41598-018-27748-9

**Published:** 2018-06-25

**Authors:** Yanyan Chen, Rosa Baños, Javier Buceta

**Affiliations:** 1 Department of Bioengineering, Lehigh University, Iacocca Hall, 111 Research Dr., Bethlehem, PA 18015 USA; 2 Barcelona Science Park, Carrer de Baldiri Reixac, 4-12-15, 08028 Barcelona, Spain; 3 Department of Chemical and Biomolecular Engineering, Lehigh University, Iacocca Hall, 111 Research Dr., Bethlehem, PA 18015 USA

## Abstract

Regardless of the progress achieved during recent years, the mechanisms coupling growth and division to attain cell size homeostasis in bacterial populations are still not well understood. In particular, there is a gap of knowledge about the mechanisms controlling anomalous growth events that are ubiquitous even in wild-type phenotypes. Thus, when cells exceed the doubling size the divisome dynamics sets a characteristic length scale that suggests a *sizer* property. Yet, it has been recently shown that the size at birth and the size increment still satisfy an *adder*-like correlation. Herein we propose a Markov chain model, that we complement with computational and experimental approaches, to clarify this issue. In this context, we show that classifying cells as a function of the characteristic size set by the divisome dynamics provides a compelling framework to understand size convergence, growth, and division at the large length scale, including the adaptation to, and rescue from, filamentation processes. Our results reveal the independence of size homeostasis on the division pattern of long cells and help to reconcile *sizer* concepts at the single cell level with an *adder*-like behavior at a population level.

## Introduction

Cell size is a major trait that determines the cellular physiology and is regulated by the coordination of growth and division. Thus, given specific metabolic conditions, cells display “narrow” size distributions that are maintained over generations and indicate the existence of size control mechanisms^1–4^. The simplest approach to understand size control is the 1-to-2 rule: given that for every cell that has doubled its size to divide two daughter cells are generated, then one would expect to find that the fraction of cells of size “1” doubles the fraction of cells with size “2”^5^. Yet, experiments reveal a more complex scenario where cells display either smaller or larger sizes, and in some cases even extreme anomalous growth (e.g. filamentation in bacteria), and yet achieve homeostasis^6–10^.

Different hypotheses have been then proposed exploring the idea that space and/or time regulation underlies size homeostasis. The *sizer* model assumes that cells are able to “measure” distances: cells grow until division is triggered if their length excedes a size, *l*_*d*_, which is uncorrelated with the cell size at birth, *l*_*b*_. In this regard, researchers have shown that yeast satisfies the sizer hypothesis and in, both, budding and fission yeast, there are control points in their cell cycles based on their sizes^11–14^. More recently, it has been also suggested that cell differentiation in plants is driven by a sizer mechanism^15^. On the other hand, in the *timer* model the cells are assumed to be able to “measure” time: cells grow until division is triggered when the cell cycle duration surpasses a threshold^16,17^. While currently discussed, *C*. *crescentus* bacteria seem to follow a timer mechanism to control division^9,18^. In these studies, researchers have profited from high-throughput techniques that allow to obtain and analyze large sets of data at the single cell level^19^. Interestingly, it has been shown that rod-shaped bacteria, e.g. *E*. *coli*, contradict both the sizer and timer hypotheses. Instead, these bacteria satisfy the so-called *adder*, aka *incremental*, model, such that cells add a constant increment to their size at birth before division, Δ*l* = *l*_*d*_ − *l*_*b*_, that, on average, is independent of *l*_*b*_^8,9,20,21^. Such rule, when coupled with a symmetric division process, leads to size convergence and homeostasis. Thus, the reported independence of the increased size Δ*l* on the size at birth *l*_*b*_ apparently falsifies the sizer hypothesis^9,22^ and the size-dependent generation time is not compatible with the timer model either^9,23^. Additionally, it has been suggested that the correlation observed between *l*_*d*_ and *l*_*b*_ appeals to the idea that memory effects could play a role in size regulation and homeostasis^24^. However, experimental results argue in favor of a memoryless mechanism^8^.

On the modeling side, Osella *et al*. have proposed a stochastic phenomenological modeling to introduce the idea that size is not the only variable controlling cell division, and the time spent in the cell cycle also plays a critical role^23^. Other modeling approaches using stochastic arguments have shown theoretically that the adder, sizer, and timer models can be unified under a general paradigm by adjusting an interpolation factor^16,25^, and that the lognormal-like size distribution can be obtained from the master equation formalism when halving and growth are considered^26^. Taheri-Araghi and coworkers proposed different models (sizer, timer, and adder) considering Poissonian statistics for the splitting rate and reviewed their predictions showing that the adder model is the only one fully consistent with the experimental observations^9^. Finally, some models have tried to establish a relation between cell size regulation and the hypothetical activity of membrane proteins regulating division as the underlying mechanism of the sizer behavior^20,27^.

**Figure 1:**
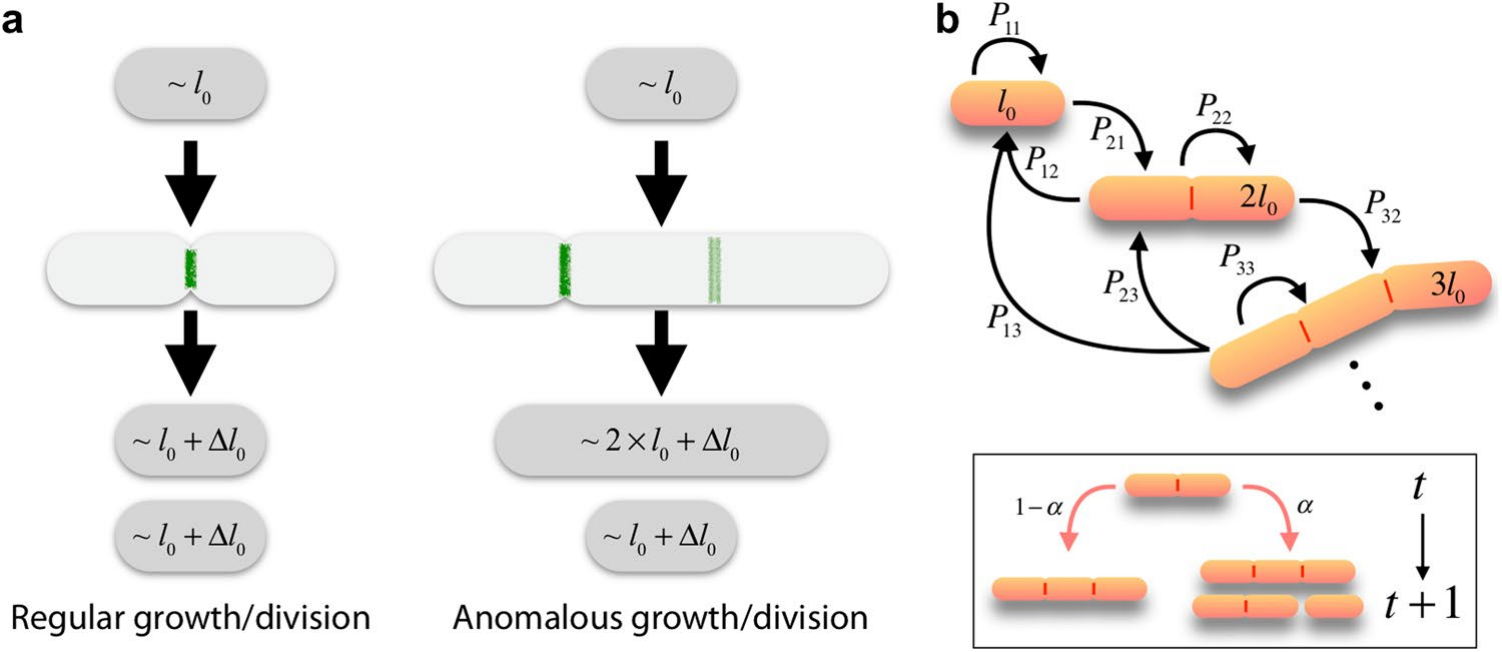
(**a**) In regular growth/division events the relevant fluctuations in the size increment are of order Δ*l*_0_; however, in anomalous growth/division events the dominant ones are of order *l*_0_. The green lines indicate either the actual (dark) or putative (light) division sites. (**b**) Our model, by neglecting small scale fluctuations in size changes, assumes that the length of a cell is well represented by the number of units *l*_0_ contained in a cell and establishes probabilities for the transitions between those states, ${P}_{nm}\,:\,=\,{P}_{m\to n}$ (from *m* to *n*), that depend on the division pattern (see text). Inset: the efficiency of the division machinery is described by means of the parameter *α*: cells either grow with probability 1 − *α* or grow/divide with a probability *α* (division is considered to happen instantaneously).

However, besides all these advances, the mechanisms coupling growth and division to achieve cell size homeostasis are still not well understood. In particular, while most of the efforts have focused on understanding how fluctuations around the typical cell size are shaped to achieve size homeostasis, there is a gap of knowledge about the contribution of cell growth/division at a larger length scale, i.e. when cells grow three-fold longer, or more, than their typical size, see Fig. 1a. In that regard, a recent study in the context of cell filamentation has suggested that the adder principle may have a wider applicability and poses the interesting problem of making it compatible with a sizer behavior that quantizes the division locations^28^. Moreover, this problem also applies to wild-type cells, since subpopulations that deviate from typical sizes are ubiquitous and yet size homeostasis is achieved. In this context, current knowledge about the division mechanisms in rod-shaped bacteria indicates that the septum location is determined by the interactions between division proteins, the Min oscillatory system, and the nucleoid occlusion machinery. Here, for simplicity, we refer to these set of mechanisms that synergistically contribute to set a characteristic length of cells as the divisome dynamics. In particular, in *E*. *coli* the septum precursor, FtsZ, localizes where the spatiotemporal-averaged concentration of its inhibitor MinC is minimal and sets a characteristic length in cells arguably driven by a Turing-like mechanism^29–35^. Under regular conditions, only one septum is formed by the middle of the cell (longitudinal axis). However, if division fails and cells grow longer, multiple rings are formed at regular intervals along the cell thus determining multiple, putative, cleavage sites^36^. This poses the intriguing question of understanding what is the influence of the division pattern for setting a cell size distribution and for enabling size convergence. In fact, experimental results on filamentous phenotypes have shown that an adder-like correlation is satisfied regardless of the propensity of long cells to undergo asymmetric divisions^20,28^. In relation to this, experiments on ΔminC strains have shown that the positioning of the septum is not relevant at the short length scale either to satisfy the adder correlation^8^.

Here we focus on these questions and, by combining theoretical, computational, and experimental work, we address the problem of understanding how large size fluctuations (of the order of the characteristic length set by the divisome dynamics and larger) contribute to size regulation. Our study provides a framework to describe how cells adapt to, and are rescued from, filamentation processes, and allows to characterize the division efficiency of rod-shaped bacteria. In this context, we show how size convergence can be achieved and demonstrate that it is independent of the division pattern followed by multiple division sites. Our study helps to reconcile concepts and shows that, at the large length scale, a sizer feature at the single-cell level, i.e. to consider a well defined length scale associated with division events, is compatible with the experimental observations about the adder correlations at the population level.

## Results

### Modeling Framework: a Markov Chain Model

The division machinery sets a typical cell size *l*_0_. Thus, in a regular growth/division process a size increment of the order *l*_0_ + Δ*l*_0_ is achieved, where Δ*l*_0_ accounts for the variability around the characteristic length *l*_0_, i.e. the relative fluctuations in the growth of daughter cells with respect the mother cell are of order Δ*l*_0_/*l*_0_, Fig. 1a. Previous studies have focused on how these fluctuations are shaped to achieve size convergence. However, the reported existence of cells with lengths longer than “1” or “2” *l*_0_ units under balanced growth conditions, or in filamentation processes, indicates that the division machinery is not 100% effective. In these anomalous growth/division events the dominant relative fluctuations in growth/division events are of order *l*_0_ due to discretization of the cleavage locations, Fig. 1a. However, the ratio Δ*l*/〈*l*_*b*_〉 still achieves a value close to one at the level of the population average, see^20,28^.

Here we argue that in those growth events the small fluctuations can be neglected because they are superseded by the cleavage discretization effects and the possible sizes of daughter cells can be described by the combination of discrete units, *l*_0_ (Fig. 1b). While in a different context, this discretization idea is in line with recent results that suggest that the cell size is the sum of the initiation mass (i.e. the cell size at replication initiation per origin)^37^. Yet, we point out that the unit size, as defined by division machinery, refers to a different concept. In particular, the initiation mass is invariant under perturbations while the unit size, *l*_0_ depends on the growth conditions (see below). In our model we further assume that, under steady state growth conditions, the time required by a cell to increase its size an amount *l*_0_ is well defined, *t*_0_, and we describe the temporal status of a cell by means of snapshots in units of *t*_0_ (yet, ultimately this time does not contribute to the characteristics of the stationary size distribution). In addition, we assume that *t*_0_ is larger than the cleavage timescale and we consider that the latter occurs instantaneously. Finally, in order to account for the probabilistic nature of the division machinery (the division efficiency), we introduce a parameter, *α*, that recapitulates the factors that control the commitment of cells towards division. Notice that in our approach we do not consider cell death since the apoptotic rate during the lag/exponential growth phases (our main focus) is negligible. Altogether, we propose a probabilistic discrete space-and-time approach to describe the cell growth and division processes in rod-shaped cells; i.e. a Markov chain modeling framework. Our approach is then alike to the one proposed to study the packing and topological organization in epithelial monolayers^38^. Our model has several limitations, being unable to describe the continuous growth process the most noticeable (see Discussion). In addition, our model cannot capture size fluctuations at the short length scale. Yet, it offers some advantages and novelties, such as allowing to explore the adaptation to, and the rescue from, filamentation, the role played by the division pattern and asymmetric cleavage, and to reconcile sizer concepts linked to the divisome dynamics with results obtained from an adder principle.

By denoting by *p*_*m*_ the probability of a cell to have a “size” (length) $m\in {{\mathbb{Z}}}^{+}$ (in units of *l*_0_) at “time” $t\in {\mathbb{Z}}$ (in units of *t*_0_), the cell size distribution is then characterized by the vector ${\bf{p}}(t)={({p}_{1},{p}_{2},{p}_{3},\ldots ,{p}_{N})}^{T}$ and its temporal dynamics is prescribed by,

1 $${\bf{p}}(t+1)={\mathbb{P}}\times {\bf{p}}(t)$$

where ${\mathbb{P}}$ stands for the (*N* × *N*)-transition matrix whose elements, *P*_*nm*_, are the probabilities of obtaining a cell of size *n* at “time” *t* + 1 given that either that cell or its mother had a size *m* at “time” *t* (see Fig. 1b). Note that our model tacitly assumes that filamentous cells increase their size linearly. This approximation can be justified experimentally (Fig. S1, see also^28^). In addition, we point out that the filamentation speed does not dictate the statistics of cell sizes. Note that *p*_*m*_ can be interpreted as the fraction of cells in a population having a size *m* and *P*_*nm*_ as the fraction of cells “transitioning” from size *m* to size *n* per unit of time.

We consider that ${\mathbb{P}}$, and hence *P*_*nm*_, is separable depending on the effectiveness of the division machinery such that,

$${P}_{nm}=(1-\alpha ){P}_{nm}^{g}+\alpha {P}_{nm}^{g+d}$$

where ${P}_{nm}^{g}$ and ${P}_{nm}^{g+d}$ stand for the growth and the growth + division probabilities respectively, and 0 ≤ *α* ≤ 1 is the probability of a division event when a cell increases its length by *l*_0_ (we disregard the possibility of multiple simultaneous division events), see Fig. 1b. That is, on one hand, ${P}_{nm}^{g}$ represents the probability of a cell to reach a size *n* at time *t* + 1 by *growing* from a size *m* at time *t*. Thus, ${P}_{nm}^{g}={\delta }_{n,m+1}$, where *δ*_*i*,*j*_ stands for the Kronecker delta. On the other hand, ${P}_{nm}^{g+d}$ accounts for the probability of obtaining a cell of size *n* at time *t* + 1 by *dividing* a cell that grew from a size *m* at time *t*. In that regard, we first notice that given a cell of size *m* at time *t*, its size at time *t* + 1 becomes *m* + 1 and, following an “instantaneous” (single) division event, two daughter cells of sizes *n* and *m* + 1 − *n* are born. Each of the two daughter cells will have a size of, at least, one (*l*_0_) unit; thus, only *m* + 1 − 2 = *m* − 1 units need to be distributed among daughter cells. Consequently, to estimate ${P}_{nm}^{g+d}$ reduces to the probability problem of “picking” *n* − 1 elements from a set with *m* − 1 elements.

Assuming that all prospective septa are equiprobable, such probability (probable cases divided by possible cases) reads $$(\begin{array}{c}m-1\\ n-1\end{array})/{2}^{m-1}$$, where $$(\begin{array}{c}m\\ n\end{array})=\frac{m!}{n!(m-n)!}$$ is the binomial coefficient. Thus,

2 $${P}_{nm}=(1-\alpha ){\delta }_{n,m+1}+\alpha (\begin{array}{c}m-1\\ n-1\end{array})\frac{\theta (m+1-n)}{{2}^{m-1}}$$

where *θ*(*z*) is the Heaviside theta function: *θ*(*z*) = 1 if *z* > 0 (0 otherwise).

Note that the fact that the prospective septa are equiprobable does not imply that the daughter cell sizes satisfy an uniform distribution. In order to understand the influence of the division pattern here we explore other cases such as a division by the middle pattern,

3 $${P}_{nm}^{g+d}={\delta }_{\widehat{(m/2)},1}\,{\delta }_{n,\frac{m+1}{2}}+{\delta }_{\widehat{(m/2)},0}\frac{{\delta }_{n,\frac{m}{2}}+{\delta }_{n,\frac{m+2}{2}}}{2}$$

where $\hat{\cdot }$ indicates the modulo operation, an inverse binomial distribution favoring divisions close to the cell poles,

4 $${P}_{nm}^{g+d}={\delta }_{n,1}\,{\delta }_{m,1}+(1-\frac{(\begin{array}{c}m-1\\ n-1\end{array})}{{2}^{m-1}})\frac{\theta (m+1-n)}{m-1}$$

and finally a uniform probability for growth and division,

5 $${P}_{nm}^{g+d}=\frac{\theta (m+1-n)}{m}$$

As for the relation between size and division pattern, we expect that a subpopulation of *l*_0_-sized cells will just double their size, up to 2 × *l*_0_, and then divide. In a wild-type phenotype, where *α* is expected to be close to 1, this cell population is in fact the largest. In those cases a symmetric division at the middle is the only possible case regardless of the division pattern. For this regular growth/division events our modeling approach reproduces, trivially, the expected population average behavior for size increments, i.e. Δ*l*/〈*l*_*b*_〉 = 1; however, the model cannot capture the processes leading to size convergence due to the fluctuations around *l*_0_.

It is easy to check that if the maximum size a cell can have is unbounded, i.e. if *N* → ∞, then ${\sum }_{n=1}^{\infty }\,{P}_{nm}=1$, that is, ${\mathbb{P}}$ is properly normalized. However, and while to the best of our knowledge a precise quantification is missing, the unconstrained growth of cells seems unrealistic and a maximal cutoff value for *N* constitutes a more reasonable hypothesis, that is, cells cannot grow indefinitely^39,40^. In that case we notice that ${\sum }_{n=1}^{N}\,{P}_{nN}=\alpha$, i.e. the normalization condition for ${\mathbb{P}}$ breaks down for the maximum cell size state. To address this issue, we assume that *α* = 1 in that case: once a cell reaches the maximal length it cannot grow anymore and should either remain with that size or divide. That is, for example in the case of the binomial division pattern, $${P}_{nN}=(\begin{array}{c}N-1\\ n-1\end{array})\frac{1}{{2}^{N-1}}\forall n$$.

The stationary cell size distribution is reached when **p**(*t* + 1) = **p**(*t*) = **p**^*st*^ (i.e. the fraction of cells with a given size does not change with time). By implementing this condition in Eq. (1) and imposing the normalization of the probability vector, **p**^*st*^ can be obtained by solving the following linear system of equations: $({\mathbb{I}}-{\mathbb{P}})\times {{\bf{p}}}^{st}=0\cup {\sum }_{n=1}^{N}\,{p}_{n}^{st}=1$ (${\mathbb{I}}$ being the *N* × *N* identity matrix).

### Theoretical Analysis: Characterization of the Growth-Division Dynamics

The features and predictions of our modeling approach with respect to the growth-division dynamics can be characterized as follows. First, once the stationary probabilities **p**^*st*^ are estimated the probability of *new* born cells with a given size *n* reads,

6 $${\pi }_{n}=\sum _{m=n}^{N}\,{p}_{m}^{st}{P}_{nm}$$

Note that ${p}_{n}^{st}$ can be written as a function of *π*_*n*_ as,

7 $${p}_{n}^{st}={\pi }_{n}+\theta (n-1)\,\sum _{m=1}^{n-1}\,{\pi }_{m}\,\prod _{k=m}^{n-1}\,{P}_{(k+1)k}$$

We notice that since at any particular time a fraction of cells are growing then ${\sum }_{n=1}^{N}\,{\pi }_{n} < 1$. That is, the fraction of growing cells reads $(1-{\sum }_{n=1}^{N}\,{\pi }_{n})$.

As a function of *π*_*n*_ two relevant quantities can be estimated. On the one hand, the stationary probability of a cell to have an initial size *n*, ${\pi }_{n}^{\ast }$, that we can compute by collecting all the events for which cells were born with an initial size *n* and did not undergo division yet,

8 $${\pi }_{n}^{\ast }={\pi }_{n}(1+\sum _{m=n+1}^{N}\,\prod _{k=n}^{m-1}\,{P}_{(k+1)k})$$

On the other hand, the probability of a cell to grow from a length *m* at birth and divide when the size has increased by *n* > 0 units reads,

9 $${p}_{m,n}^{\ast }=(\alpha +{\delta }_{n,N-m}(1-\alpha ))\,{\pi }_{m}{(1-\alpha )}^{n-1}\,\theta \,(N-m)\,\theta \,(N-m+1-n)$$

We stress that ${p}_{m,n}^{\ast }$ does not satisfy a normalization condition: ${\sum }_{n=1}^{N}\,{p}_{m,n}^{\ast } < 1$ (not all cells that increase their length are born with size *m*) and, additionally, ${\sum }_{m=1}^{N}\,{p}_{m,n}^{\ast } < 1$ (not all cells that are born increase their size *n* units).

### Size Convergence and the Adder Principle: Numerical Simulations

Our theoretical approach allows us to compute analytically the dimensionless average length increment of cells, 〈*n*〉_*m*_, that are born with (dimensionless) size *m* (i.e. Δ*l* = *l*_0_〈*n*〉_*m*_, and *l*_*b*_ = *l*_0_*m*),

10 $${\langle n\rangle }_{m}=\frac{{\sum }_{n=1}^{N}\,n{p}_{m,n}^{\ast }}{{\sum }_{n=1}^{N}\,{p}_{m,n}^{\ast }}=\frac{1-{(1-\alpha )}^{N-m}}{\alpha }$$

Interestingly, since the probabilities *π*_*m*_ trivially cancel out when computing 〈*n*〉_*m*_, this implies that the length increment is independent of the properties of the division process, that is, it is independent of the division pattern. However, the statistical properties of the length distributions, e.g. size at birth, do depend on the division pattern. This might explain the independence of the size increment in strains that supposedly have the same division efficiency but different septa positioning^8^.

The validity of the well-known adder relation, Δ*l*/〈*l*_*b*_〉 = 1 regardless of the value of *l*_*b*_, can be analyzed by estimating the deviation of the average increment from a constant value. To that end, we compute the slope of the average length increment, 〈*n*〉_*m*_, as a function on *m* (size at birth) at the “origin” (the smallest length at birth registered),

11 $$\delta n={\frac{\partial {\langle n\rangle }_{m}}{\partial m}|}_{m=1}=\frac{{(1-\alpha )}^{N-1}\,\mathrm{ln}\,(1-\alpha )}{\alpha }$$

In case an adder-like correlation holds then ${\langle n\rangle }_{m}\simeq {\rm{const}}{\rm{.}}$ and consequently $\delta n\simeq 0$. Equation (11) indicates that *δn* = 0 in the non-trivial limit: *N* → ∞. In that case ${\langle n\rangle }_{m}={\rm{const}}.=1/\alpha$ (i.e. Δ*l* = *l*_0_/*α*).

Thus, our model predicts that as long $N\gg 1$ the average size increment is independent of the size at birth regardless of the value of the division efficiency. Indeed, experimental studies suggest that the maximum size *E*. *coli* cells can growth, *N*, can be ${\mathscr{O}}({10}^{2})$^39,40^. This behavior at cell scales larger than *l*_0_ has been confirmed in studies performed on strains with increased antibiotic sensitivity that display a filamentous phenotype, e.g. the so-called *frik* cells^20^ and, more recently, in rescue from filamentation processes^28^.

**Figure 2:**
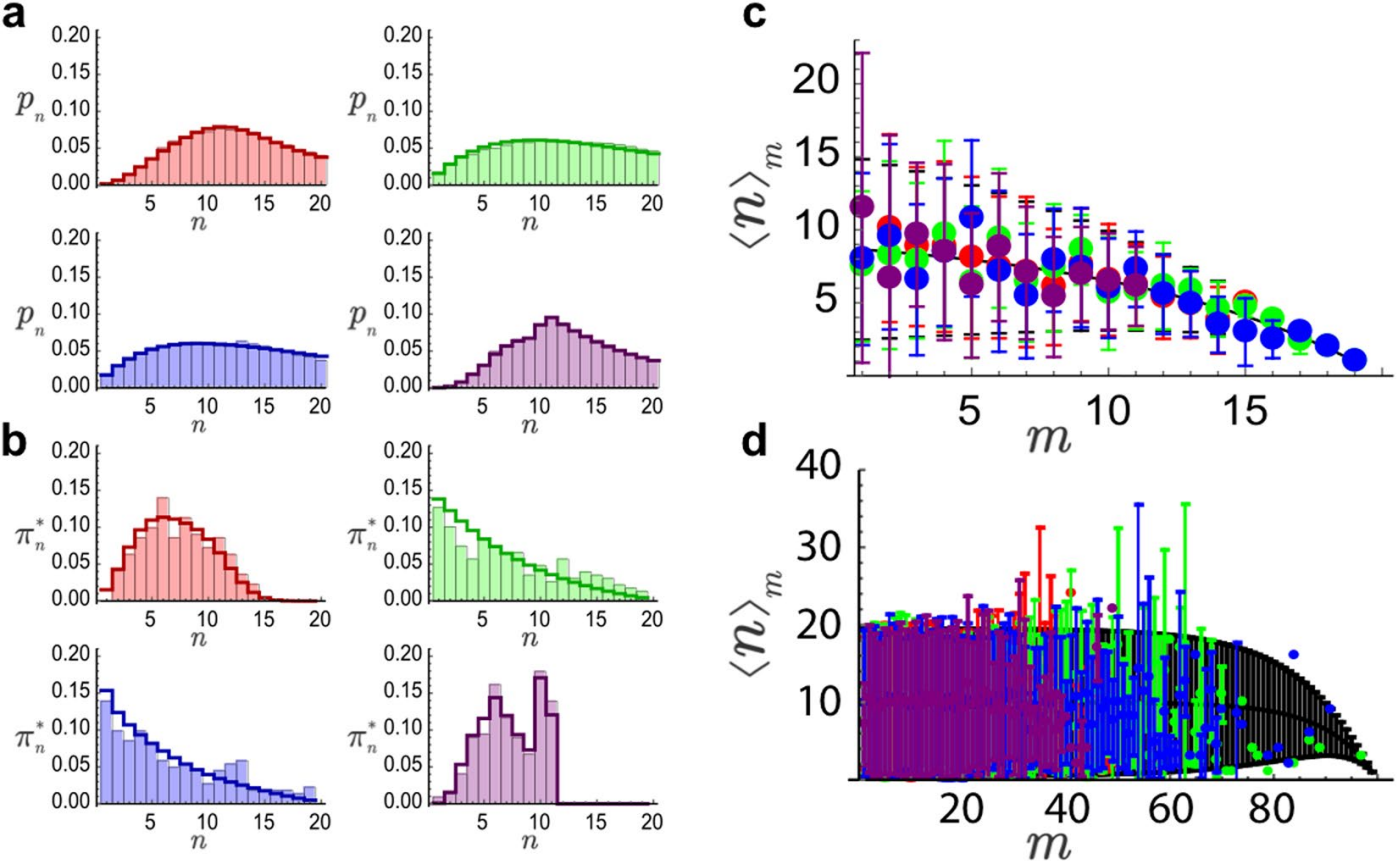
Probabilities of cell size (**a**) and cell size-at-birth (**b**) as a function of the division pattern for the case *α* = 0.1 and *N* = 20. Bars: numerical simulations; Lines: exact stationary solution. Division pattern color code: red, green, blue, and purple stand for binomial, uniform, inverse binomial, and division by the middle respectively. Notice that some lengths cannot be reached if cells divided by the middle and that the uniform and inverse binomial division patterns leads to almost indistinguishable size statistics. The panels (c and d) show that the average increment is independent of the division pattern and that finite size effects are noticeable as long as *m* ~ *N*: (**c**) *N* = 20, (**d**) *N* = 100; symbols correspond to numerical simulations (same color code as in panels (a and b)) and the black line to the theoretical solution given by Eq. (10) (error bars stand for the standard deviation).

In order to confirm these predictions, we ran computer simulations using different division patterns (Methods). Figure 2a,b shows that different division patterns produce different size statistics in filamentous phenotypes; yet, all of them achieve size homeostasis and lead to the same functional relation for 〈*n*〉_*m*_ as predicted by Eq. (10), Fig. 2c,d. Finite size effects are noticeable at length scales *m* ~ *N* and as *N* increases an adder-like correlation 〈*n*〉_*m*_ = const. = 1/*α* is recovered (Fig. 2c,d). Similar results are obtained in the case of a wild-type phenotype (*α* close to, but different that, one), Fig. S2. However, in that situation all division patterns generate, as expected, virtually indistinguishable size distributions.

**Figure 3:**
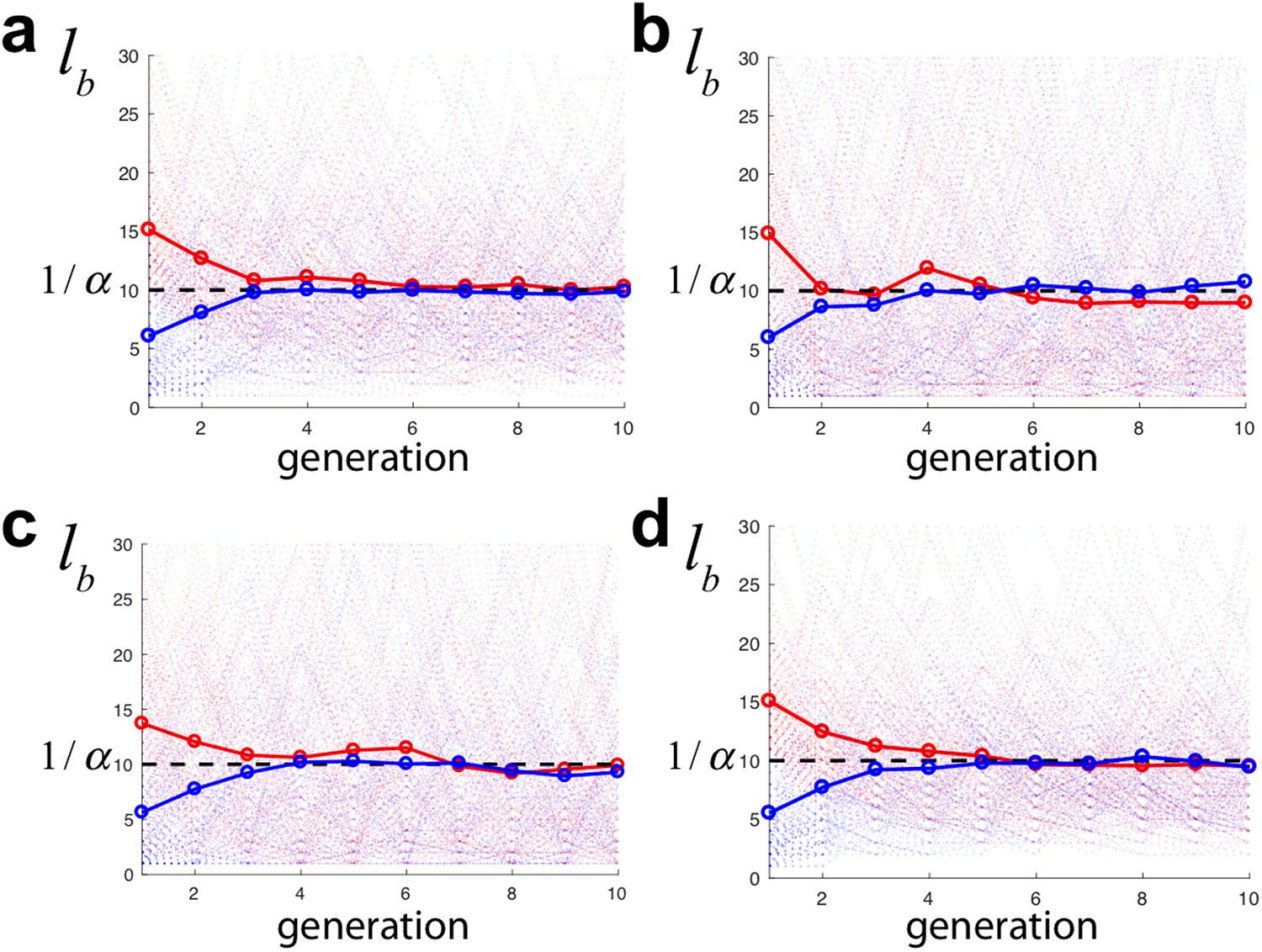
Size at birth *l*_*b*_ (in units of *l*_0_) as a function of the cell generation (numerical simulations, 150 trajectories). In all cases *α* = 0.1. The light colors stand for individual trajectories and solid colors for the average. Red and blue trajectories indicate cells with an initial condition *l*(*t*_0_) = 20*l*_0_ and *l*_0_ respectively. (**a–d**) Panels stand for different division patterns: binomial, uniform, inverse binomial, and division by the middle respectively. In all cases size convergence is achieved after few generations such that 〈*l*_*b*_〉 = 1/*α* = Δ*l*.

Our simulations also confirmed that, neglecting finite size effects, the following relation holds regardless of the division pattern, 〈*l*_*b*_〉 = *l*_0_/*α* (i.e. 〈*m*〉 = 1/*α*), see Fig. S3. Consequently, our model predicts that the size increment Δ*l* = 〈*l*_*b*_〉 (i.e. 〈*n*〉_*m*_ = 〈*m*〉) in agreement with all previous studies. We stress that in our model we do not impose an adder principle, i.e. cells are not forced to increment their size with respect to the length at birth by a given, fixed, amount. Yet, cells show size convergence and satisfy an adder-like correlation. Thus, in our model the latter and size homeostasis behavior cannot be explained as a consequence on the adder principle as in previous modeling approaches. Our results further argue that the “adder correlation” is compatible with a sizer principle that sets a characteristic length scale, *l*_0_. Interestingly, our simulations show that our model leads to size convergence in processes adapting to, or being rescued from, filamentation independently of the division pattern, Fig. 3. In all cases cell sizes evolves towards the value 〈*l*_*b*_〉 = Δ*l* = *l*_0_/*α*. In the context of wild-type phenotypes, our model also accounts for size convergence; however, since we neglect the fluctuations around *l*_0_, the model fails to capture the convergence phenomenon at scales smaller than *l*_0_ (Fig. S4).

### Estimation of the Division Efficiency and the Division Pattern: Experiments

**Figure 4:**
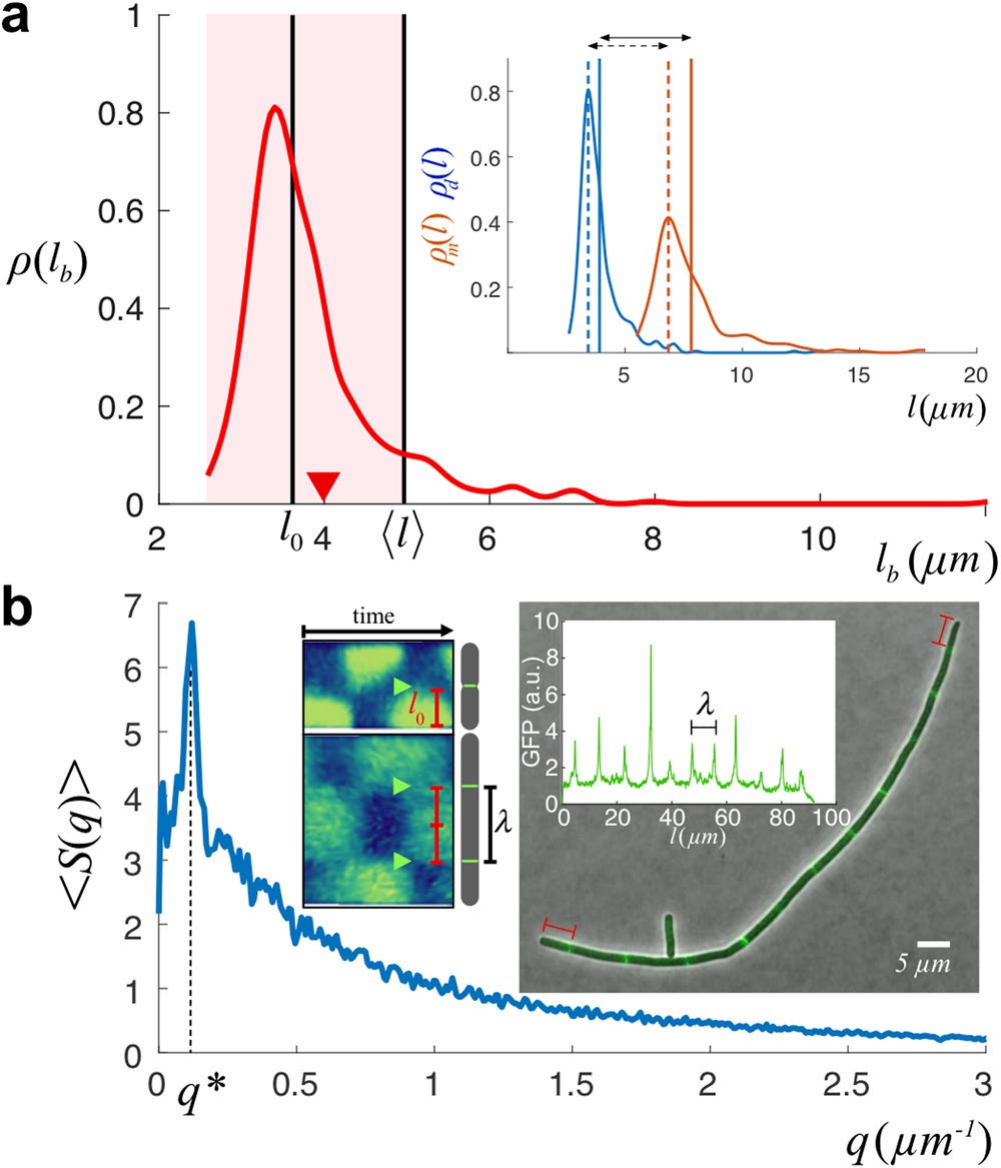
(**a**) The cell size at birth distribution, *ρ*(*l*_*b*_), was used as a proxy for determining *l*_0_. We filter the data discarding cells with a size of birth larger than the average plus one standard deviation (i.e. cells with a Z-score larger than one): the shadow region indicates the data considered after filtering to estimate *l*_0_. The black vertical line shows the computed value of *l*_0_ = 3.6 ± 0.5 *μm* and its comparison with 〈*l*〉 = 5 ± 2 *μm* (40% larger than the unit division length *l*_0_). The red triangle indicates the value of 〈*l*_*b*_〉 = 4 *μm* before filtering out filamentous cells. The inset shows additional measurements to estimate the unit division length: by computing the histograms of sizes of mother, *ρ*_*m*_(*l*), and daughter, *ρ*_*d*_(*l*), cells (244 and 488 single cell data points respectively) the unit division length *l*_0_ can be estimated as the difference between the most probable value of their sizes (dotted lines, ~3.3 *μm*) or by the differences of the averages (solid lines, ~3.9 *μm*). (**b**) The modulus of the averaged power spectrum 〈*S*(*q*)〉 (a.u.) of the fluorescent signal of filamentous cells (MGZ202 in LB, 165 cells) reveals a peak at q* = 0.12 *μm*^−1^ and indicates the regularity of the septum-septum distance: *λ* = 1/q* = 8.3 *μm*. In filamentous cells, the distance between consecutive Z-rings, as a proxy for the characteristic minimal size upon division, is 2 × *l*_0_ due to the doubling in size of central Min domains as cells grow longer as revealed by the kymograph (inset, adapted from^35^): *l*_0_ = *λ*/2 = 4.2 *μm*. The Z-ring organizes at locations where the averaged spatiotemporal concentration of Min is minimal (arrows). This phenomenon may also be the reason underlying the preferential asymmetric division of filamentous cells close to the poles during sporadic cleavage events (Fig. S9). The right-most inset shows a sample of a filamentous cell and the GFP fluorescent intensity signal (FtsZ expression).

In order to test some of the predictions of our model, we measured the growth and division dynamics of wild-type *E*. *coli* cells under exponential growth conditions (see Methods, Fig. S5). Our modeling approach neglects the fluctuations around *l*_0_ and assumes that a discrete sampling of cell lengths as a function of the unit size *l*_0_ leads to a good representation of the otherwise continuous size distribution. Thus, in order to compare with theoretical expressions, we performed a clustering process, i.e. a classification scheme of cells, where the cell size is binned as a function of *l*_0_. To characterize the unit cell size, *l*_0_, we used the stationary probability of the cell sizes at birth, *ρ*(*l*_*b*_), that we computed by tracking division events in time-lapse movies (Movie S1, Fig. S5), and filtered out filamentous cells, and cells transitioning towards filamentation, by discarding the tail of the distribution. We set the cut-off length to be 〈*l*_*b*_〉 + *σ*; where 〈*l*_*b*_〉 = 4 *μm* and *σ* = 1 *μm* are the mean (red triangle Fig. 4a) and the standard deviation of the full distribution respectively, i.e. we discarded cells with a Z-score larger than one. This statistical method for filtering out long cells is akin to that used in other studies to select cells with a filamentous phenotype^19^. Here we single out the opposite, i.e. “short” cells. According to this, cells with a Z-score between one and two are then considered to be “transitioning” to a filamentous phenotype.

Once long cells have been discarded, we estimated *l*_0_ by computing the average of the distribution: *l*_0_ = 3.6 ± 0.5 *μm* (Fig. 4a). Thus, Δ*l*_0_/*l*_0_ ~ 0.14 and, as mentioned above, the fluctuations around *l*_0_ are negligible with respect to the variability in size increments due to the divisome dynamics. In order to test the reliability of our approach, we also computed *l*_0_ using the size statistics of mother and daughter cells. On one hand, we estimated *l*_0_ as the difference of the most probable values of their lengths, ~3.3 *μm*. Alternatively, we quantified *l*_0_ as the difference between their means, ~3.9 *μm* (Fig. 4a). We notice that the estimation using the means includes the information of cells transitioning to filamentation and consequently can be considered as an upper bound. Finally, we expect *l*_0_ to be a readout of the underlying cellular processes that set a characteristic, minimal, size of cells to be viable (divisome dynamics). Thus, we checked that our estimation of *l*_0_ is compatible with the measurement of the distance between consecutive FtsZ rings (Fig. 4b). Since wild-type cells only present a single Z-ring, we developed a strain, *E*. *coli* MGZ202, with a filamentous phenotype that contains a FtsZ:GFP fusion thus showing different prospective septa (see Methods). In order to check if *l*_0_ varies under different nutrient conditions, we performed our measurements using LB and M9 minimal media (Methods). A Fourier analysis of the FtsZ:GFP fluorescent signal indicated that *l*_0_ = *λ*/2 = 4.2 *μm* in LB and 3.9 *μm* in M9, a ~7% decrease (Fig. S6). Assuming that the division efficiency does not depend on the media, this shrinkage of *l*_0_ as the nutrient level decreases is in agreement with previous experimental results^41^. Altogether, the different quantifications of the characteristic unit cell size in LB provide consistently a value around 4 *μm*. Hereafter, we assume *l*_0_ = 3.6 *μm* with a variability of 0.5 *μm* as determined by the measurements of the cell sizes at birth in the wild-type phenotype since it has the largest statistics.

Once we determined *l*_0_ we obtained the experimental values of the probabilities *p*_*n*_ and ${\pi }_{n}^{\ast }$ by a binning procedure. We clustered the experimental data from the length and length-at-birth stationary probability distributions, *ρ*(*l*) and *ρ*(*l*_*b*_) respectively, using boxes of size 1 (in units of *l*_0_) centered at *l*\*l*_0_ (where\indicates integer division),

12 $${p}_{n}=\frac{1}{{l}_{0}}\,{\int }_{{l}_{0}(n-\frac{1}{2})}^{{l}_{0}(n+\frac{1}{2})}\,\rho (l)\,dl$$

13 $${\pi }_{n}^{\ast }=\frac{1}{{l}_{0}}\,{\int }_{{l}_{0}(n-\frac{1}{2})}^{{l}_{0}(n+\frac{1}{2})}\,\rho ({l}_{b})\,dl$$

We noticed that the classification scheme only makes sense if the relative size of the fluctuations Δ*l*_0_/*l*_0_ is small. In this way, the classification scheme is able to distinguish among cell classes in terms of their *l*_0_-size. While we acknowledge the existence of fluctuations in *l*_0_, critical to achieve size convergence at the “short” length scale, they don’t play a critical role in the results obtained from the classification procedure (see below sensitivity analysis).

**Figure 5:**
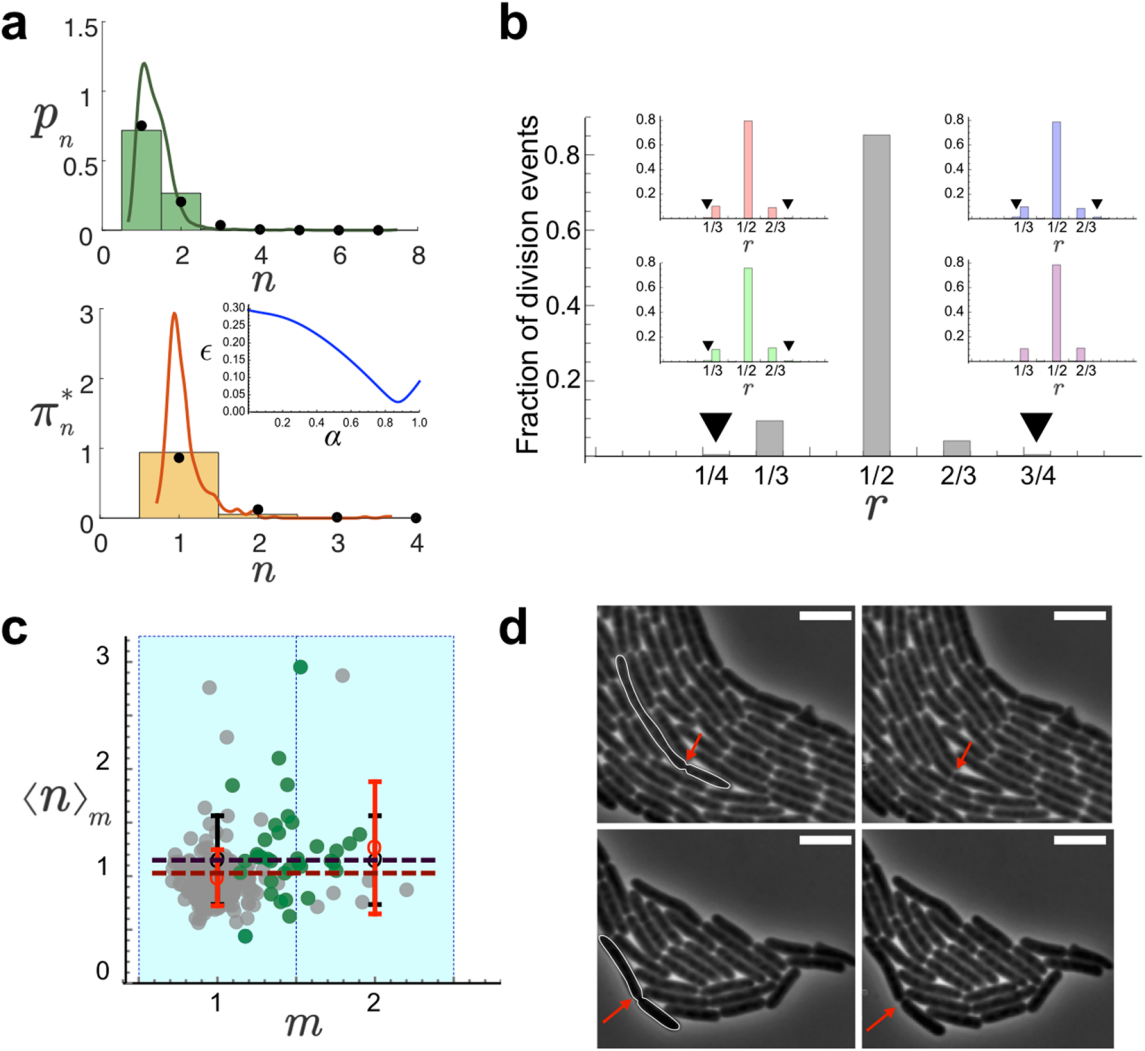
(**a**) Stationary probabilities of cell sizes *p*_*n*_ (top, 3297 single cell data points) and cell sizes at birth ${\pi }_{n}^{\ast }$ (bottom, 488 single cell data points) binned as a function of the state *n* (*l*_0_ units). The lines correspond to the unbinned, continuous, distributions *ρ*(*l*) (top) and *ρ*(*l*_*b*_) (bottom). The clustering process defines cell classes depending on the units *l*_0_ contained and allows to compare theoretical predictions and experimental results. By fitting simultaneously the experimental probabilities to the theoretical values (inset shows error minimization as a function of *α*) we estimated the value of the division efficiency, *α* = 0.87, when the division pattern considered was all septa to be equiprobable (binomial pattern of division): black circles. Alternative division patterns produce similar results (Fig. S7). (**b**) Fraction of division events as a function of the ratio, *r*, between mother and daughter cell sizes as obtained experimentally. The insets show the results of numerical simulations for different division patterns using the value of *α* as measured in experiments: red, green, blue, and purple stand for binomial, uniform, inverse binomial, and division by the middle respectively. The black triangles point out 1/4 and 3/4 division fractions, barely visible, that reveal the existence of cell sizes up to 4 unit lengths. All tested division patterns but the division by the middle show the 1/4 and 3/4 division ratios. (**c**) Size increment as a function of the length at birth (both in units of *l*_0_). Points stand for single cell data (252). Grey points (216) account for cells born from symmetric divisions. Green points (36, including one outlier at *m* = 4 not shown in the figure) indicate cells born from asymmetric divisions (~15% of the division events). Open red circles stand for binned experimental values, open black circles for numerical simulations using the values of *l*_0_ and *α* as experimentally measured. The black line corresponds to the theoretical estimate, Eq. (10) using *N* = 20 and *α* = 0.87. The red line corresponds to the fitting of the experimental binned points to the theoretical estimate 1/*α*: *α* = 1.0 ± 0.1. The error bars stand for the standard deviation. (**d**) Snapshots showing examples of asymmetric division events. Time runs from left to right. 5 *μm* scale bar in all cases. The outlined cells on the left divide and generate two cells satisfying 1/4–3/4 (top) and 1/3–2/3 (bottom) size relations. The red arrows indicate the location of the septa.

Figure 5a shows the output of the binning procedure. Our results provide a simplified, but compelling, perspective of the growth and division process that indicates that in wild-type cells three populations, in terms of their *l*_0_ size, account for ~99% of the size distribution and ~10% of cells grow beyond 2 × *l*_0_. Thus, as expected, the cell size probability at homeostasis showed deviations from the 1-to-2 rule, indicating the presence of “3” (and even longer) units cells, and therefore failures of the division machinery, i.e. *α* < 1. The classification scheme additionally allows us to check the consistency of our estimation of *l*_0_ using liquid cultures. We notice that in liquid cultures *l*_0_ cannot be obtained from the size-at-birth distribution or the mother-daughter statistics since we cannot possibly register time-lapse movies in that growth medium. However, we argue that the division efficiency of wild-type *E*. *coli* cells, i.e. their intrinsic commitment towards division, is the same in liquid and solid cultures. According to our modeling approach, size probabilities (normalized histograms) in the steady state can be collapsed in a master “curve” by rescaling them by their corresponding *l*_0_’s if the *α* values are the same. We then obtained the cell size probability in liquid cultures (Methods) and implemented a minimization of the error, $\epsilon$, as a function of *l*_0_ (in liquid), where ${\epsilon }^{2}=\frac{1}{N}\,{\sum }_{n}\,{({p}_{n}{|}_{{\rm{solid}}}-{p}_{n}{|}_{{\rm{liquid}}})}^{2}$. Our result, Fig. S6, showed that the histograms collapse when *l*_0_ = 3.5 *μm* (error ~0.2%) suggesting that *l*_0_ does not depend on growing the cells in liquid or solid media as long as the nutrient composition is equivalent.

A ballpark figure of *α* can be obtained from the relation predicted by our model: *α* = *l*_0_/〈*l*_*b*_〉 = 0.9 ± 0.2. In order to obtain a more precise quantification of the division efficiency, we performed a double regression of the experimental data (solid cultures) against theoretical expressions and minimized the error function, $\epsilon$, where ${\epsilon }^{2}$ = $\frac{1}{N-1}\,{\sum }_{n}\,{({\pi }_{n}^{\ast }{|}_{\exp .}-{\pi }_{n}^{\ast }{|}_{{\rm{theo}}.})}^{2}$ + $\frac{1}{N}\,{\sum }_{n}\,{({p}_{n}{|}_{\exp .}-{p}_{n}{|}_{{\rm{theo}}.})}^{2}$ (Fig. 5a). That is, we fit simultaneously the size and the size at birth distributions to their corresponding theoretical expressions. The value found when using a binomial division pattern was *α* = 0.87 ± 0.03 and confirmed the large, yet limited, division efficiency of wild-type cells. The experimental data tested against other division patterns yielded the same value for *α* with the exception of the division the middle pattern that gave *α* = 0.88 (Fig. S7). Given that our theoretical approach assumes a constant value of *l*_0_, we test the robustness of our results with respect to the variability of *l*_0_ by means of a sensitivity analysis. Since the variability of *l*_0_ is smaller than the bin size we expect that the cell classification does not change significantly when the fluctuations are considered. Consequently the variability observed in the estimation of *α* should not increase, but decrease, indicating a robust behavior of our scheme against *l*_0_ fluctuations. In this regard, when we repeated the estimation of *α* (binomial division pattern) using *l*_0_ = 3.1 *μm* (average minus one standard deviation) we obtained *α* = 0.78 ± 0.05. On the other hand, when we computed the division efficiency using *l*_0_ = 4.1 *μm* (average plus one standard deviation) we got *α* = 0.94 ± 0.01. Thus, the variability (coefficient of variation) of the output, (*α* value) 13%, was smaller than the variability of the input, (*l*_0_ value) 20%, and evidence the robustness of our results.

In agreement with the theoretical analysis and simulations (Fig. S1), when *α* ~ 1 it was not possible to distinguish among different division patterns since the probability of getting cells with sizes larger than four (in units of *l*_0_) is very low and under those conditions all tested division pattern produced almost identical distributions since most cleavage processes are symmetric. Only when cells start developing a filamentous phenotype they undergo asymmetric divisions (Fig. 4c,d)^20^ (see Discussion). To further confirm this point we computed the ratio, *r*, between the (binned) sizes of mother and daughter cells and tested it against different division patterns by means of simulations. Figure 5b shows that the experimental data was compatible with all tested division patterns but the division by the middle since on top of the ratios 1/2, 1/3, and 2/3 the ratios 1/4 and 3/4 (barely noticeable but existent, see Fig. 5c,d) were also registered in experiments and those are not compatibles with that division mode. This test also provided additional evidence of the existence of, mainly, three cell populations in wild-type cells in terms of their *l*_0_ size. For the sake of quantitatively determining the division pattern we re-analyzed recent data of cells recovering from filamentation processes^28^ using additional statistical tools: Kolmogorov-Smirnov (KS) test, see Fig. S8. The results of KS test showed maximum compatibility with the inverse binomial and the uniform division distributions and, to a lesser extent, with the binomial distribution. In addition, in agreement with the analysis of wild-type cells, the data rejected the possibility that cells divide preferentially by the middle when different cleavage sites are formed. This analysis confirmed our own observations of the filamentation process in the strain MGZ202 that displayed cleavage preferentially by the cell tips (Fig. S9).

Finally, we tested if the value obtained of the division efficiency was compatible with our predictions about the adder correlation: 〈*n*〉_*m*_ = 1/*α* (i.e. Δ*l* = *l*_0_/*α*). We stress that, as mentioned above, our model cannot capture the role of small fluctuations for size convergence but we argue that those are not relevant for the functional dependence of the correlation at the population level since ${\rm{\Delta }}{l}_{0}/{l}_{0}\ll 1$. According to the value obtained for *α* we expect 〈*n*〉_*m*_ = 1.15. Figure 5c shows that our results are in agreement with these predictions.

## Discussion

Herein we have presented a Markov model to understand the growth and division dynamics of rod-shaped bacteria that focuses on anomalous growth processes. Our approach differs from those proposed in the field so far since it is coarse-grained and disregards small fluctuations around *l*_0_. Instead, we study the behavior in situations where the fluctuations at larger length scales, of the order of the characteristic length set by the divisome dynamics, *l*_0_, and dominant. Consequently, our model cannot account for the variability that has been found to be key to understand size convergence when cells follow a regular growth/division pattern, Fig. 1 ^9^. However, our model provides useful information to understand how cells adapt to, and recover from filamentation processes, to clarify how the experimentally reported cleavage discretization and the division pattern contributes to size homeostasis, and to quantify the commitment towards division by estimating their division efficiency. In addition, our study sheds light into the reasons that make compatible a sizer property due to the divisome dynamics and the observed correlation between size increments and the length at birth at the collective level that is a trademark of an adder behavior. Importantly, we do not assume an adder principle and yet we have demonstrated that size homeostasis can be achieve regardless of the division pattern and an adder-like correlation is satisfied. Thus our model can explain previous experimental results that have revealed that the relation Δ*l* = 〈*l*_*b*_〉 is also observed in filamentous phenotypes where an asymmetric division pattern is evident^20,28^. Our model has additional limitations that restrict its applicability as a general formalism. In particular, the discretization of space and time precludes to address any property related to continuous growth, as for example the dependence of size on the chromosome replication cycle^37,42^. Rather, our model can be seen as discrete snapshots of the growth/division process.

The existence of a well-defined, constant, unit size, *l*_0_, is a fundamental hypothesis of our model. Still, our data show some degree of variability, Δ*l*_0_. We have neglected that variability on the basis of the small value observed for the ratio Δ*l*_0_/*l*_0_. The performed sensitivity analysis shows that the classification scheme and the value obtained for the division efficiency of wild-type cells are robust with respect the fluctuations of *l*_0_. The cell discretization concept, while different, is in line with recent studies revealing the existence on an invariant unit size at replication initiation per origin^37^. This rises the intriguing question of a possible connection between these concepts. Here we have provided different, consistent, ways to estimate *l*_0_ including its indirect quantification through the distance between prospective septa. Notably, the analysis confirms, indirectly, the apparent doubling in size of the central Min domains as cells grow longer^35^. The latter indicates another limitation of our model since we have assumed that septa are always separated apart by a distance *l*_0_. This seems to be the case for cells with a length up to ~3 × *l*_0_. In longer cells, only the distance between pole and neighboring Z-ring seems to be of size *l*_0_. We argue that this might explain the preferential division of filamentous cells by the poles.

Our experiments confirm the high, yet limited, capability of wild-type cells to successfully proliferate (*α* ~ 0.9) but data, when questioned about the underlying division pattern, apparently show compatibility with all tested distributions due to low percentage of events, ~10%, leading to cells longer than 3 × *l*_0_ in that phenotype. However, our analysis of recent experimental data obtained from cells recovering from filamentation^28^, supports that cells divide following either an inverse binomial distribution that favors cleavage by the tips when several septa are formed or an uniform distribution. Such lack of distinctness between these two distributions is in agreement with our simulations, Fig. 2. Further experiments providing more statistics would be eventually able to settle this issue and might even reveal a changing pattern as a function of the cells’ length. Also, the analysis rejects categorically the possibility of a division by the middle in cells longer than 2*l*_0_ and strongly suggest that cells do not follow a binomial division pattern.

As a matter of discussion, here we have assumed that there is a growth limitation in terms of size. Recent studies have revealed that in fact *E*. *coli* cells cannot grow indefinitely and limiting sizes of ~${\mathscr{O}}({10}^{2}\,\mu m)$ have been reported in cells for which the division was artificially blocked^39,40^. This connects with our assumption about the rule that applies to the last state. In that regard, we have assumed that cells either remain in that state or divide, but other options are certainly possible; for example, to introduce a death probability. However, the lack of recurrence of the last state implies that it does not contribute significantly to the size statistics and, consequently, neither do the rules that are prescribed for the longest cell.

Divisome-mutant backgrounds or drug-treated cultures for which the division efficiency is significantly smaller than in a wild-type background, may further confirm some predictions of our framework. Still, we notice that testing our model in filamentous phenotypes involves technical problems difficult to overcome even using high throughput techniques; including to extrapolate to the wild-type case scenarios where the division is conditioned either genetically or by drugs. The mutant strain that we have introduced here, MGZ202, is a clear example as our observations suggest that the division efficiency of growing cells is not constant: some subpopulations grow developing a filamentous phenotype whereas some other cells apparently divide normally. Under those conditions it is not possible to compare with our modeling framework. Moreover, previous results indicated long-term memory effects during filamentation that we cannot possibly explain within our framework^19^. In this context, here we have analyzed data from a recent study that has implemented a novel approach to study recovery from filamentation and has shown, in agreement with our model, a wider applicability of an adder-like correlation^28^. Further work along those lines might shed extra light on these anomalous growth processes with interest in the context of engineering applications^39,40^.

In summary, our study, makes compatible the idea of some sizer capabilities by rod-shaped bacteria associated with the division and the nucleoid occlusion dynamics and the trademark correlations observed in cells satisfying the adder principle. Also, it provides a simple and effective framework to understand how growth and division coordinate during anomalous growth to achieve homeostasis and clarifies the influence of the division positioning and large length scale fluctuations in growth processes.

## Methods

### Construction of MGZ202 Strain (*ftsZ* Null Mutant in MG1655 Carrying an *gfp*:*ftsZ* Fusion)

MGZ202 strain is an *ftsZ* null mutant in *E*. *coli* MG1655 that carries an IPTG inducible *mut2gfp*^43^: *ftsZ* fusion in a plasmid (pGP025, Tanneke den Blaauwen lab). To obtain an *ftsZ* deletion mutant from strain *E*. *coli* MG1655 (pGP025) we used the Gene Bridges- Quick and Easy Gene Deletion Kit based on Red/ET recombination method. In order to generate a functional cassette with homology arms corresponding to the sequences flanking *ftsZ* gene plus FRT sites, we amplified by PCR the FRT-PGK-gb2-neo-FRT template DNA provided by the kit using primers FTSKM5 and FTSKM3. Primer FTSKM5 (5′ GACGATGATTACGGCCTCAGGCGACAGCACAAATCGGAGAGAAACTAT **AATTAACCCTCACTAAAGGGCG** 3′) contained 46 bp upstream the initiation codon of *ftsZ* gene plus 2 bp of the first codon. Primer FTSKM3 (5′ AGCCTCGAAACCCAAATTCCAGTCAATTCTTAATCAGCTTGCTTACGCAT **AATACGACTCACTATAGGGCTC** 3′) contained the last 33 bp of *ftsZ* coding region plus 16 bp downstream of *ftsZ* gene. Both primers contained tails corresponding to FRT-PGK-gb2-neo-FRT cassette marked in bold. In parallel, MG1655 (pGP025) was transformed with the expression plasmid pRedET (tet) provided by the kit. The resulting strain carrying both plasmids, was induced by the addition of L-arabinose to allow the expression of genes mediating Red/ET recombination, and electroporated with the FTSKM5 - FTSKM3 PCR product: 0,36% final concentration from a 10% stock solution as described in Gene Bridges kit. Only colonies carrying the replacement of *ftsZ* coding region (from nucleotides +3 to +1060) by the FRT-PGKgb2-neo-FRT cassette survived in kanamycin plates. Finally, kanamycin selection marker was removed from the chromosome by transforming the cells with FLP expression plasmid (e.g. 707-FLPe, Gene Bridges). Primers FTSZFOR (5′ CTCAATAGTTGGCTGCGAAA 3′) and FTSZREV (5′ CGGGCCAGTTTAGCACAAAG 3′) were used to check the null mutation. Strain MG1655 (pGP025) with *ftsZ* deletion was named MGZ202. Antibiotics were used at the following concentrations: ampicillin, 200 *μ*g/mL; kanamycin, 40 *μ*g/mL; tetracycline, 3 *μ*g/mL. Agarose gel electrophoresis and DNA manipulations were performed using standard procedures. For plasmid DNA isolation, PCR product purification and recovery from DNA from agarose gels GenEluteTM kits from Sigma-Aldrich were used. PCR experiments were performed in the Perkin Elmer GeneAmp PCR System 2400 according to standard protocols, using DreamTaq PCR Master Mix (ThermoFisher). No IPTG was used for the induction of the plasmid to allow leaky expression of the *gfp*:*ftsZ* fusion and force cells to develop the filamentous phenotype.

### Solid Cultures Imaging

All reagents from Sigma-Aldrich unless stated otherwise. Strains: *E*. *coli* MG1655 and MGZ202. Cells were inoculated from a −80 °C stock into 10 *mL* (25 *g*/*L*) Luria broth (LB, Miller) or (10.5 *g*/*L*) M9 minimal medium and grown overnight (O/N) at 37 °C with shaking (200 r.p.m.). Cells were refreshed in LB or M9 minimal medium (same conditions) for 2.5 *h* to ensure exponential growth (Fig. S1). The cell culture was then diluted to a final *OD*_600_ ~ 0.001 (Eppendorf Biophotometer Plus) for the sake of obtaining single-colony imaging ROIs. 2 *μL* cell samples were inoculated on solid pads made of LB medium (25 *g*/*L*) or M9 minimal medium (10.5 *g*/*L*) with low-melting agarose (2%). The pads were obtained by sandwiching 2 *mL* of the filtered (0.2 *μm*) mix @ 55 °C in 24 m*m* × 60 m*m* microscope slides, then left to solidify for 30 mins, and cut to a final size of ~0.5 *cm* × 0.5 *cm*. The inoculated pads were put in a desiccator (<25% relative humidity) for 20 mins at room temperature and then transferred to microscopy plates (IBIDI *μ*-Dish 35 mm low) with the inoculated surface of the pads facing the bottom glass of the plate. The temperature during imaging was set at 37 °C (IBIDI temperature control system).

### Liquid Cultures Imaging

#### Strain

*E*. *coli* MG1655. After the initial O/N of the frozen stock, the cell culture was diluted in fresh LB medium (25 *g*/*L*) to *OD*_600_ ~ 0.05 and cultured at 37 °C with shaking (150 r.p.m.). We collected *OD*_600_ data every 20 mins until *OD*_600_ saturated (~5 hours in 20 *mL* samples). The measured growth curve (Fig. S1) was used to determine the *OD*_600_ at which cells exited the exponential growth and started transitioning to the stationary phase: *OD*_600_ ~ 0.35. Fresh cultures were grown under similar conditions, including the O/N dilution in fresh medium (*OD*_600_ ~ 0.05), until *OD*_600_ ~ 0.35. At that point 2 *μL* samples were spotted on solid pads for imaging (following the protocol mentioned above for solid cultures) and, in addition, 1:10 dilutions samples were cultured again until *OD*_600_ ~ 0.35 to obtain new 2 *μL* samples for microscopy. We repeated the culture procedure up to 5 times to confirm steady-state growth conditions.

### Microscopy and Image Analysis

The agarose pads were imaged with an inverted microscope (Leica DMi8, AFC autofocus) using a 100×/1.40 NA objective (HC PL APO, Leica; Oil: Type F, Leica) without magnifier. We used Phase Contrast imaging under Kohler illumination conditions using a 10 m*s* exposure time. Fluorescent imaging (MGZ202): GFP (Ex: 470/40 nm, Em: 525/50 nm) filter and 75 m*s* exposure time. Excitation was performed using a led lamp (Lumencore light engine SOLA SE 5-LCR-VA). The images were registered using a CMOS camera (Orca Flash 4.0 V.2; Hamamatsu) at 65 n*m*/*pixel* resolution using 2,048 pixel × 2,048 pixel image sizes @ 16 bits (depth). In the case of solid cultures, we typically imaged 4 different positions per pad using a 2 mins time-lapse (10 m*s* exposure time) and followed cell growth for ~4 hours. In the case of liquid culture pads, since cells were already grown in exponential phase, we captured single time-point snapshots at different locations showing a large enough number of cells, ~10^2^ per ROI. Imaging conditions similar to those in solid cultures.

The software for image acquisition was Leica LAS X V.3. Image preprocessing (background subtraction) was performed using Fiji, segmentation, and quantification (including cell length measurements and GFP fluorescence intensity) were performed using the Oufti toolbox V.1^44^ (http://oufti.org/). All images were reviewed and corrected manually when required.

### Numerical Simulations

We solved numerically the equations that determine the steady-state of the Markov chain model and performed numerical simulations (single cell trajectories) using a Wolfram’s Mathematica code (Supp. Mat.). In the case of our simulations with *N* = 20, or *N* = 100, we tracked the dynamics of a single cell for ~3 · 10^3^ growth steps. We discarded the initial ~10^3^ growth steps to avoid non-steady state effects. Given that the Markov model is ergodic, the numerical simulations captured the size statistics of ~10^3^ cells at the steady state that is equivalent to the statistics we registered in the snapshots in the experiments in terms of the number of cells. In the case of simulations with *N* = 100 and *α* = 0.1 we tracked the dynamics of a single cell for ~3 · 10^5^ growth steps to allow enough statistics of division events.

### Statistical Analysis of the Division Pattern

Data from cells rescued from filamentation (data from Tans’ group^28^) were processed as follows. We collected cells’ length information once the medium that promotes filamentation was switched (from tetracycline to clear M9 minimal medium), see^28^ for details. To obtain the binned division ratio, see Fig. S8, we proceeded as in Fig. 5b. We first estimated the unit cell size, *l*_0_, from the size at birth distribution in clear medium (same approach that in Fig. 4a): *l*_0_ = 2.0 ± 0.6 *μm*. Second, we implemented a binning procedure to classify the cell sizes in terms of *l*_0_ units. Third, assuming that the mother cell size is a sum of the sizes of daughters cell, *l*_*m*_ = *l*_*daughter*1_ + *l*_*daughter*2_, we computed the division ratio, *r* = *l*_*daughter*1_/*l*_*m*_.

To implement the statistical analysis of the division pattern, we categorize the division events (4108) into classes depending on the mother size: *l*_*m*_ = 2 × *l*_0_ (3328 division events), *l*_*m*_ = 3 × *l*_0_ (108 division events), *l*_*m*_ = 4 × *l*_0_ (190 division events), *l*_*m*_ = 5 × *l*_0_ (196 division events), *l*_*m*_ = 6 × *l*_0_ (70 division events), *l*_*m*_ = 7 × *l*_0_ (48 division events), *l*_*m*_ = 8 × *l*_0_ (50 division events), *l*_*m*_ = 9 × *l*_0_ (26 division events), *l*_*m*_ = 10 × *l*_0_ (24 division events), *l*_*m*_ = 11 × *l*_0_ (20 division events), *l*_*m*_ = 12 × *l*_0_ (16 division events), *l*_*m*_ = 13 × *l*_0_ (18 division events), *l*_*m*_ = 14 × *l*_0_ (2 division events), *l*_*m*_ = 15 × *l*_0_ (0 division events), *l*_*m*_ = 16 × *l*_0_ (4 division events), *l*_*m*_ = 17 × *l*_0_ (2 division events), *l*_*m*_ = 18 × *l*_0_ (2 division events), *l*_*m*_ = 19 × *l*_0_ (2 division events), and *l*_*m*_ = 20 × *l*_0_ (2 division events). By keeping the same number of events as experimentally reported for a given size of the mother cell, we generated numerically divisions events using the different probability distributions (binomial, inverse binomial, by the middle, and uniform). We used these results to generate the numerical distributions of the division ratio. For each division pattern we computed 10^4^ numerical distributions (stochastic samples) of the division ratio. Finally, we implemented a Kolmogorov-Smirnov (KS) test to compare the level of similarity of the experimental and numerical results: for each division pattern the experimental result was tested again the 10^4^ numerical samples. Each KS test led to a *p*-value and we compute a histogram to better estimate the average *p*-value and its standard deviation. In the context of the KS test a small *p*-value suggests that it is unlikely that the data came from the same distributions, and a value equal to one indicates that the data came from identical distributions (null hypothesis). We used a standard 5% significance level, that is, if *p*-value < 0.05 the null hypothesis was rejected.

## Electronic supplementary material

Supplementary Material

Movie S1

## References

[1] Adiciptaningrum, A., Osella, M., Moolman, M. C., Lagomarsino, M. C. & Tans, S. J. Stochasticity and homeostasis in the *E. coli* replication and division cycle. *Nature Publishing Group* 1–8 (2015). 10.1038/srep18261 PMC4680914 26671779

[2] Anderson EC, Bell GI, Petersen DF, Tobey RA (1969). Cell growth and division. IV. Determination of volume growth rate and division probability. Biophysical Journal.

[3] Kubitschek HE (1969). Growth during the bacterial cell cycle: analysis of cell size distribution. Biophysical journal.

[4] Tzur A, Kafri R, LeBleu VS, Lahav G, Kirschner MW (2009). Cell Growth and Size Homeostasis in Proliferating Animal Cells. Science.

[5] Painter P, Marr A (1968). Mathematics of microbial populations. Annual review of microbiology.

[6] Soifer I, Robert L, Amir A (2016). Single-cell analysis of growth in budding yeast and bacteria reveals a common size regulation strategy. Current Biology.

[7] Robert L (2014). Division in Escherichia coli is triggered by a size-sensing rather than a timing mechanism. BMC Biology.

[8] Campos M (2014). A constant size extension drives bacterial cell size homeostasis. Cell.

[9] Taheri-Araghi S (2015). Cell-size control and homeostasis in bacteria. Current biology: CB.

[10] Amir A (2017). Is cell size a spandrel?. eLife.

[11] Murray AW, Hunt T (1993). The cell cycle: an introduction.

[12] Talia SD, Skotheim JM, Bean JM, Siggia ED, Cross FR (2007). The effects of molecular noise and size control on variability in the budding yeast cell cycle. Nature.

[13] Fantes, P. & Nurse, P. *Division timing*: *controls*, *models and mechanisms*, vol. 11 (Cambridge University Press, Cambridge, 1981).

[14] Schmoller KM, Skotheim JM (2015). The Biosynthetic Basis of Cell Size Control. Trends in Cell Biology.

[15] Pavelescu I (2018). A Sizer model for cell differentiation in Arabidopsis thaliana root growth. Molecular systems biology.

[16] Amir A (2014). Cell size regulation in bacteria. Physical Review Letters.

[17] Jun S, Taheri-Araghi S (2015). Cell-size maintenance: Universal strategy revealed. Trends in Microbiology.

[18] Iyer-Biswas S (2014). Scaling laws governing stochastic growth and division of single bacterial cells. Proceedings of the National Academy of Sciences.

[19] Wang P (2010). Robust growth of escherichia coli. Current Biology.

[20] Deforet M, Van Ditmarsch D, Xavier JB (2015). Cell-Size Homeostasis and the Incremental Rule in a Bacterial Pathogen. Biophysical Journal.

[21] Ghusinga KR, Vargas-Garcia CA, Singh A (2016). A mechanistic stochastic framework for regulating bacterial cell division. Scientific reports.

[22] Sauls JT, Li D, Jun S (2016). Adder and a coarse-grained approach to cell size homeostasis in bacteria. Current Opinion in Cell Biology.

[23] Osella M, Nugent E, Cosentino Lagomarsino M (2014). Concerted control of Escherichia coli cell division. Proceedings of the National Academy of Sciences of the United States of America.

[24] Robert L (2015). Size sensors in bacteria, cell cycle control, and size control. Frontiers in microbiology.

[25] Marantan A, Amir A (2016). Stochastic modeling of cell growth with symmetric or asymmetric division. Physical Review E - Statistical, Nonlinear, and Soft Matter Physics.

[26] Hosoda K, Matsuura T, Suzuki H, Yomo T (2011). Origin of lognormal-like distributions with a common width in a growth and division process. Physical Review E - Statistical, Nonlinear, and Soft Matter Physics.

[27] Fantes P, Grant W, Pritchard R, Sudbery P, Wheals A (1975). The regulation of cell size and the control of mitosis. Journal of Theoretical Biology.

[28] Wehrens M (2018). Size laws and division ring dynamics in filamentous escherichia coli cells. Current Biology.

[29] Sullivan SM, Maddock JR (2000). Bacterial division: Finding the dividing line. Current Biology.

[30] Lenz P, Søgaard-Andersen L (2011). Temporal and spatial oscillations in bacteria. Nature reviews. Microbiology.

[31] Fange D, Elf J (2006). Noise-induced min phenotypes in *e. coli*. PLoS computational biology.

[32] Huang KC, Meir Y, Wingreen NS (2003). Dynamic structures in escherichia coli: spontaneous formation of mine rings and mind polar zones. Proceedings of the National Academy of Sciences.

[33] Bonny M, Fischer-Friedrich E, Loose M, Schwille P, Kruse K (2013). Membrane Binding of MinE Allows for a Comprehensive Description of Min-Protein Pattern Formation. PLoS Computational Biology.

[34] Howard M, Kruse K (2005). Cellular organization by self-organization: mechanisms and models for Min protein dynamics. The Journal of cell biology.

[35] Walsh JC, Angstmann CN, Duggin IG, Curmi PM (2015). Molecular interactions of the min protein system reproduce spatiotemporal patterning in growing and dividing escherichia coli cells. PloS one.

[36] Addinall SG (1996). FtsZ ring formation in fts mutants. These include: FtsZ Ring Formation in fts Mutants. Journal of bacteriology.

[37] Si F (2017). Invariance of initiation mass and predictability of cell size in Escherichia coli. Current Biology.

[38] Gibson MC, Patel AB, Nagpal R, Perrimon N (2006). The emergence of geometric order in proliferating metazoan epithelia. Nature.

[39] Jahnke JP, Terrell JL, Smith AM, Cheng X, Stratis-Cullum DN (2016). Influences of Adhesion Variability on the “Living” Dynamics of Filamentous Bacteria in Microfluidic Channels. Molecules (Basel, Switzerland).

[40] El-Hajj ZW, Newman EB (2015). An escherichia coli mutant that makes exceptionally long cells. Journal of bacteriology.

[41] Taheri-Araghi, S., Bradde, S. & Sauls, J. Cell-Size Control and Homeostasis in Bacteria. *Current Biology* 385–391 (2015). 10.1016/j.cub.2014.12.009 PMC4323405 25544609

[42] Wallden M, Fange D, Lundius EG, Baltekin Ö, Elf J (2016). The Synchronization of Replication and Division Cycles in Individual E. coli Cells. Cell.

[43] Cormack BP, Valdivia RH, Falkow S (1996). Facs-optimized mutants of the green fluorescent protein (gfp). Gene.

[44] Paintdakhi A (2016). Oufti: an integrated software package for high-accuracy, high-throughput quantitative microscopy analysis. Molecular microbiology.

