# Supplementary material for: A Markovian Approach towards Bacterial Size Control and Homeostasis in Anomalous Growth Processes
Source: Sci Rep. 2018 Jun 25;8:9612. doi: 10.1038/s41598-018-27748-9 (PMC6018433; doi:10.1038/s41598-018-27748-9)
Supplement: Supplementary file 1 — Supplementary Material [file 41598_2018_27748_MOESM1_ESM.pdf]

A Markovian Approach towards Bacterial Cell Size Control and Homeostasis in Anomalous Growth Processes
(*Supplementary Material*)

Yanyan Chen[1], Rosa Baños[2], and Javier Buceta[1,3,*]

[1]*Department of Bioengineering, Lehigh University,*

Iacocca Hall, 111 Research Dr., Bethlehem, PA 18015, USA

[2]*Barcelona Science Park, Carrer de Baldiri Reixac, 4-12-15, 08028 Barcelona, Spain and*

[3]*Department of Chemical and Biomolecular Engineering, Lehigh University,*

Iacocca Hall, 111 Research Dr., Bethlehem, PA 18015, USA

[*]*Corresponding author: jbuceta@lehigh.edu*

SUPPLEMENTARY FIGURES

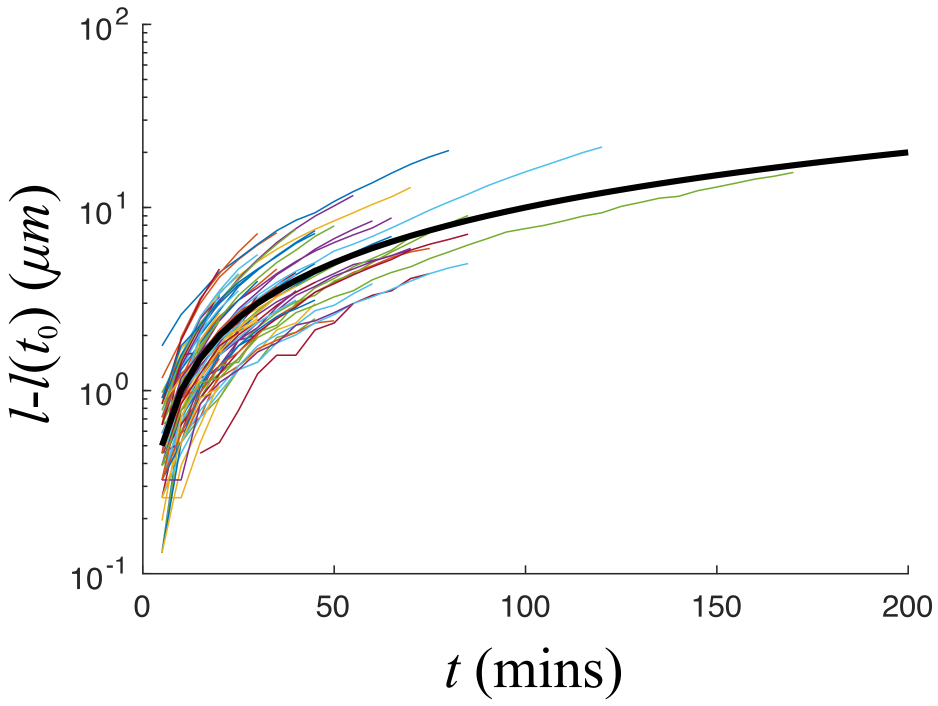

Figure S1. Cell length as a function of time during anomalous growth. The thin color lines account for individual growth events from cell birth up to division (MGZ202 strain). The wide solid black line stands for the reference curve $l - l\,(t_0) = t/10$. We used a linear-logarithmic scale to better stress the deviation from an exponential growth. Cell lengths increase linearly and only by the end of the growth process some cells display exponential growth.

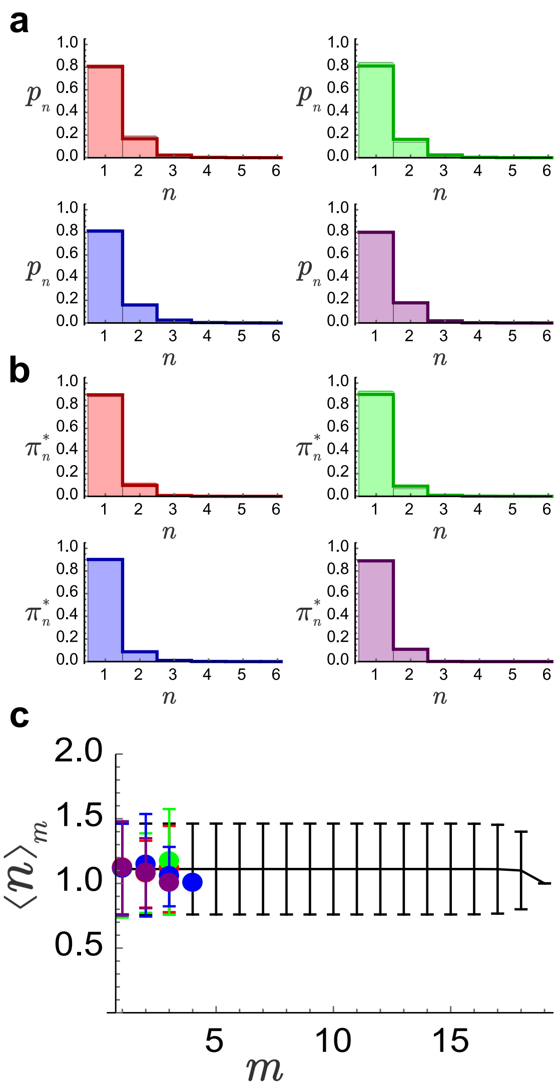

Figure S2. Probabilities of cell size (**a**) and cell size-at-birth (**b**) as a function of the division pattern for the case $\alpha = 0.9$ and $N = 20$. Bars: numerical simulations; Lines: exact stationary solution. Division patterns color code: red, green, blue, and purple stand for binomial, uniform, inverse binomial, and division by the middle respectively. In the case that $\alpha \sim 1$ the probability distributions are alike and it is not possible to distinguish among division patterns. Differences become visible, especially for the length at birth, as the division efficiency decreases. Panels **c** shows that ethe average increment is independent of the division pattern: symbols correspond to numerical simulations (same color code as in panels **a**-**b**) and the black line to the theoretical solution (error bars stand for the standard deviation). The numerical simulations when $\alpha \sim 1$ do not extend as much as the exact solution since cell sizes longer than three (in l_0 units) are highly improbable and the time for reaching those states is much longer than the simulation time.

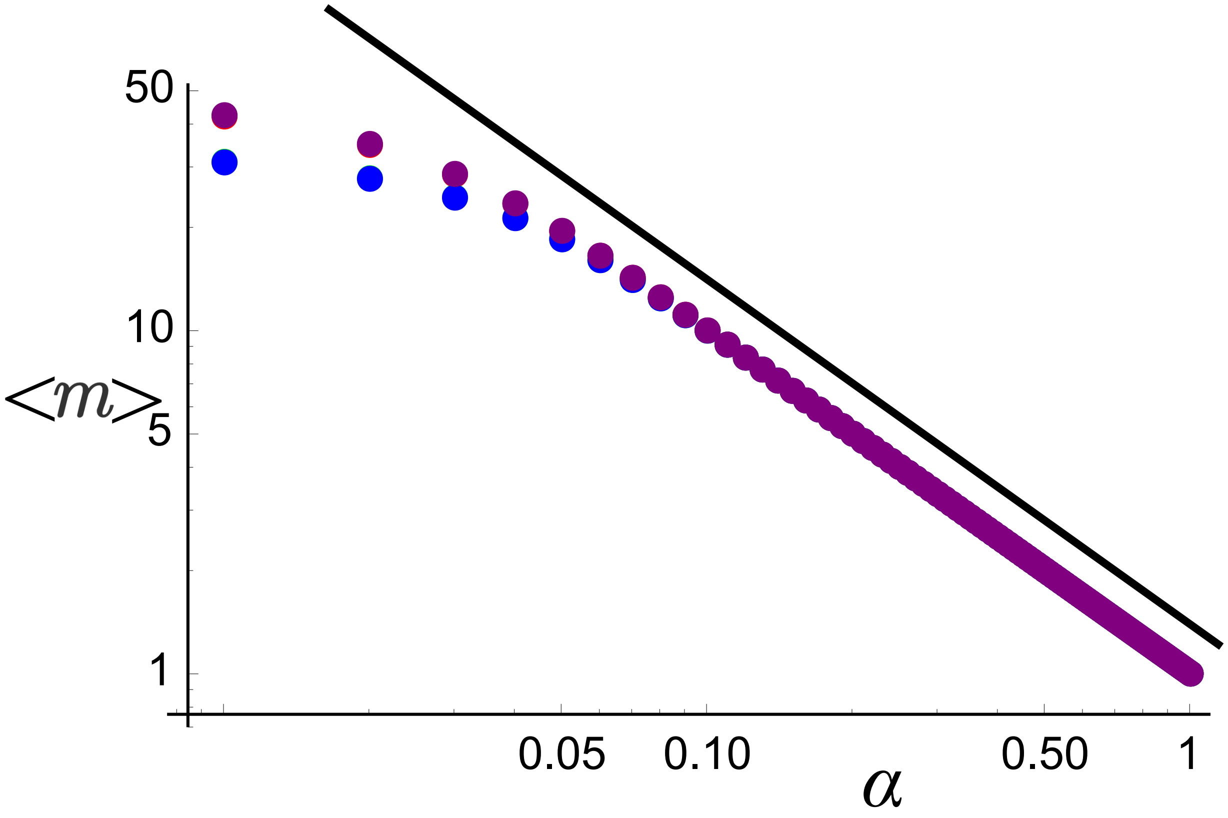

Figure S3. $\langle l_b \rangle$ (in units of l_0) as a function of α (log-log axes) for different division patterns and $N = 100$. Division pattern color code: red, green, blue, and purple stand for binomial, uniform, inverse binomial, and division by the middle respectively. The solid black line has a slope -1, i.e. $\langle l_b \rangle = l_0/\alpha$ ($\langle m \rangle = 1/\alpha$). As α decreases finite size effects (finite N) are noticeable.

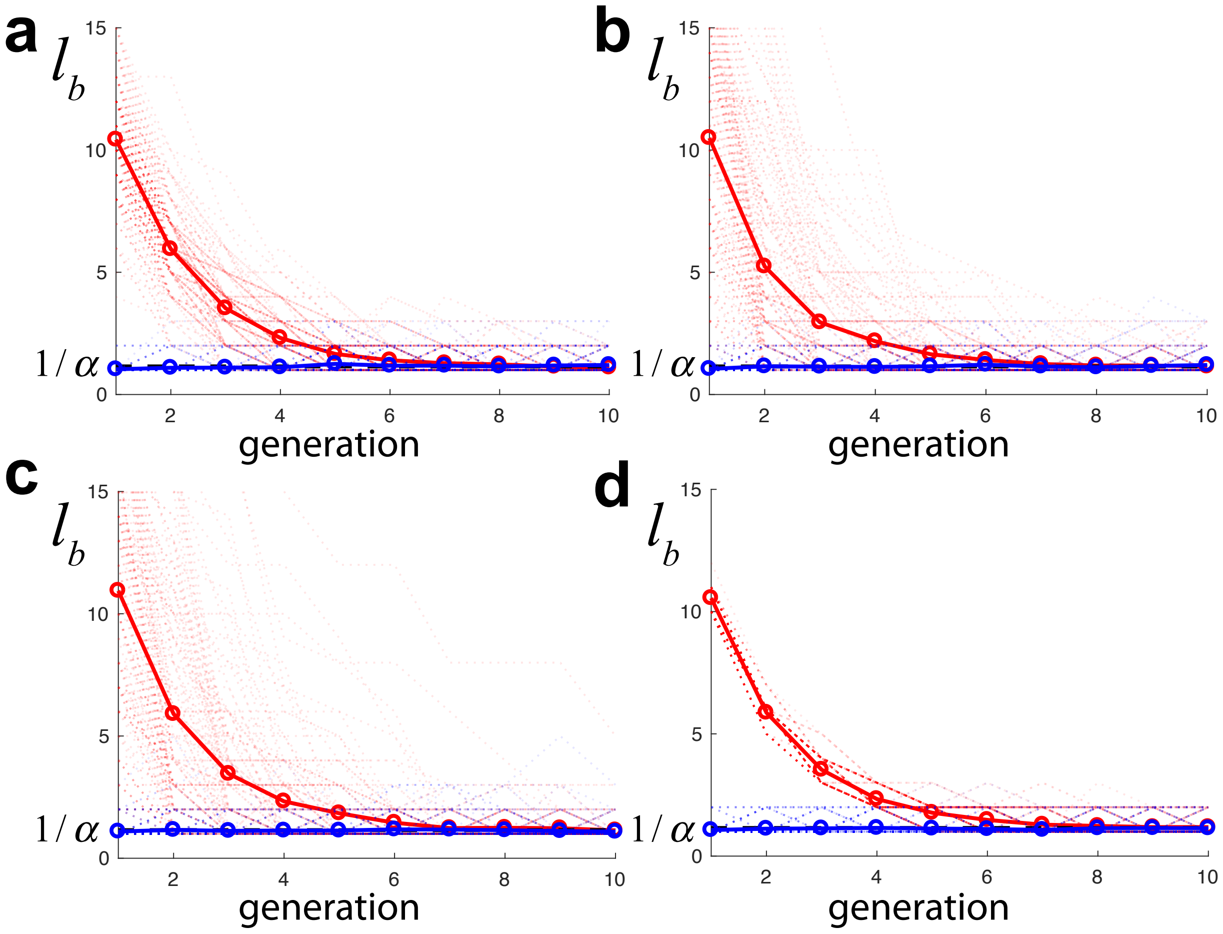

Figure S4. Size at birth l_b (in units of l_0) as a function of the cell generation (numerical simulations, 150 trajectories). In all cases $\alpha = 0.87$. The light colors stand for individual trajectories and solid colors for the average. Red and blue trajectories indicate cells with an initial condition $l(t_0) = 20l_0$ and l_0 respectively. **a-d** panels stand for different division patterns: binomial, uniform, inverse binomial, and division by the middle respectively. Size convergence is achieved after few generations for long cells such that $\langle l_b \rangle = 1/\alpha = \Delta l$. In the case of small cells our approach cannot capture convergence (i.e. it is already at the convergence regime) since it disregards small fluctuations around l_0.

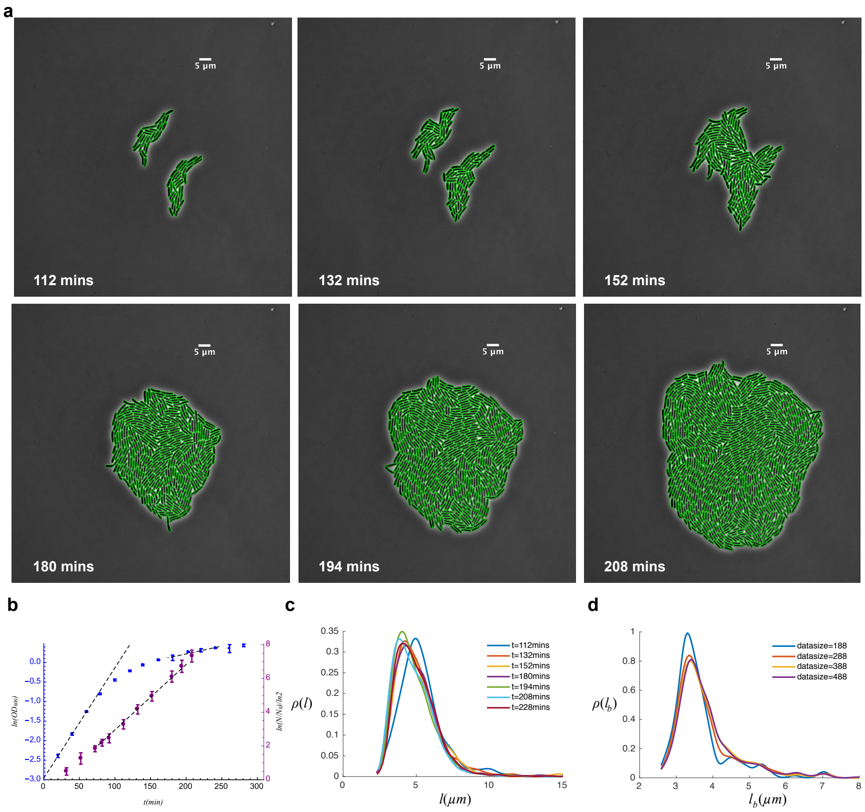

Figure S5. **a:** Snapshots of growing micro-colony (*E.coli* MG1655). The microscopy phase-contrast images (segmentation using Oufti toolbox, see Methods) show the growth during ~ 100 mins. **b:** Growth curves in liquid and solid media. **c-d:** Time and data size (number of cells) required to reach the steady state (*E.coli* MG1655). **b:** Data points with error bars in blue represent averages and STDs of $ln(OD_{600})$ in liquid cultures. The growth curve captures the exponential and early-stationary phases. The transition point between these regimes, $OD_{600} \sim 0.6$, can be estimated by the intersection of two fitting lines. The slope of the fitting of exponential phase data is ~ 0.028 indicating an average cell cycle duration of ~ 25 mins. Data points with error bars in purple represent averages and STDs of $ln(N/N_0)/ln2$ in solid culture, where N stands here for the number of cells in micro-colonies and N_0 their initial number of cells. According to an exponential growth model, $N = N_0 2^{t/\langle\tau\rangle}$, where τ represents the cell cycle duration. The fitting of the slope, ~ 0.039, indicates an average cell cycle duration of ~ 26 mins. **c:** Cell size distribution $\rho(l)$ as a function of time in solid cultures. Each time collected data from four micro-colonies. The steady state is reached after ~ 2 hours. **d:** Cell size at birth distribution $\rho(l_b)$ as a function of the data size at the steady state. A stationary profile is obtained beyond ~ 300 division events.

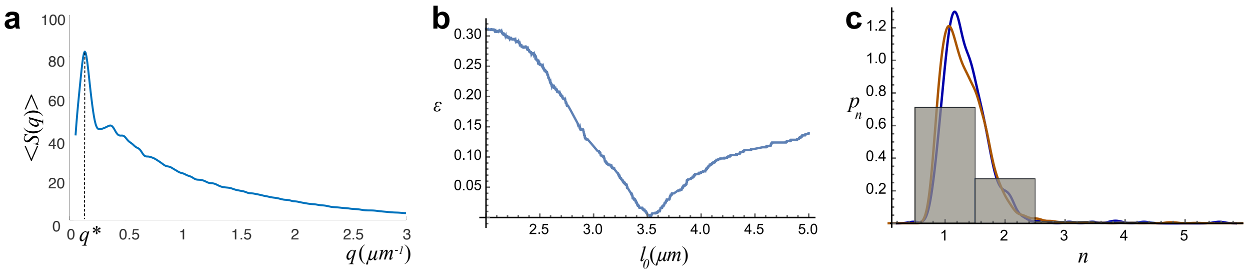

Figure S6. **a:** The modulus of the averaged power spectrum $\langle S(q) \rangle$ (a.u.) of the fluorescent signal of filamentous cells (MGZ202 in M9 minimal medium, 862 cells) reveals a peak at $q^* = 0.13\mu m^{-1}$: $l_0 = 3.9\mu m$ (a $\sim$ 7% decrease with respect to LB medium). **b:** Collapse error, ϵ, as a function of l_0 in the liquid culture. The error reaches the minimum at $l_0 = 3.5\mu m$. **c:** Collapsed, binned, stationary cell size probabilities from solid (data size 3297) and liquid cultures (data size 477), *E.coli* MG1655, LB. Lines are the unbinned, continuous distributions $\rho(l)$ of the solid (orange) and liquid (blue) cultures.

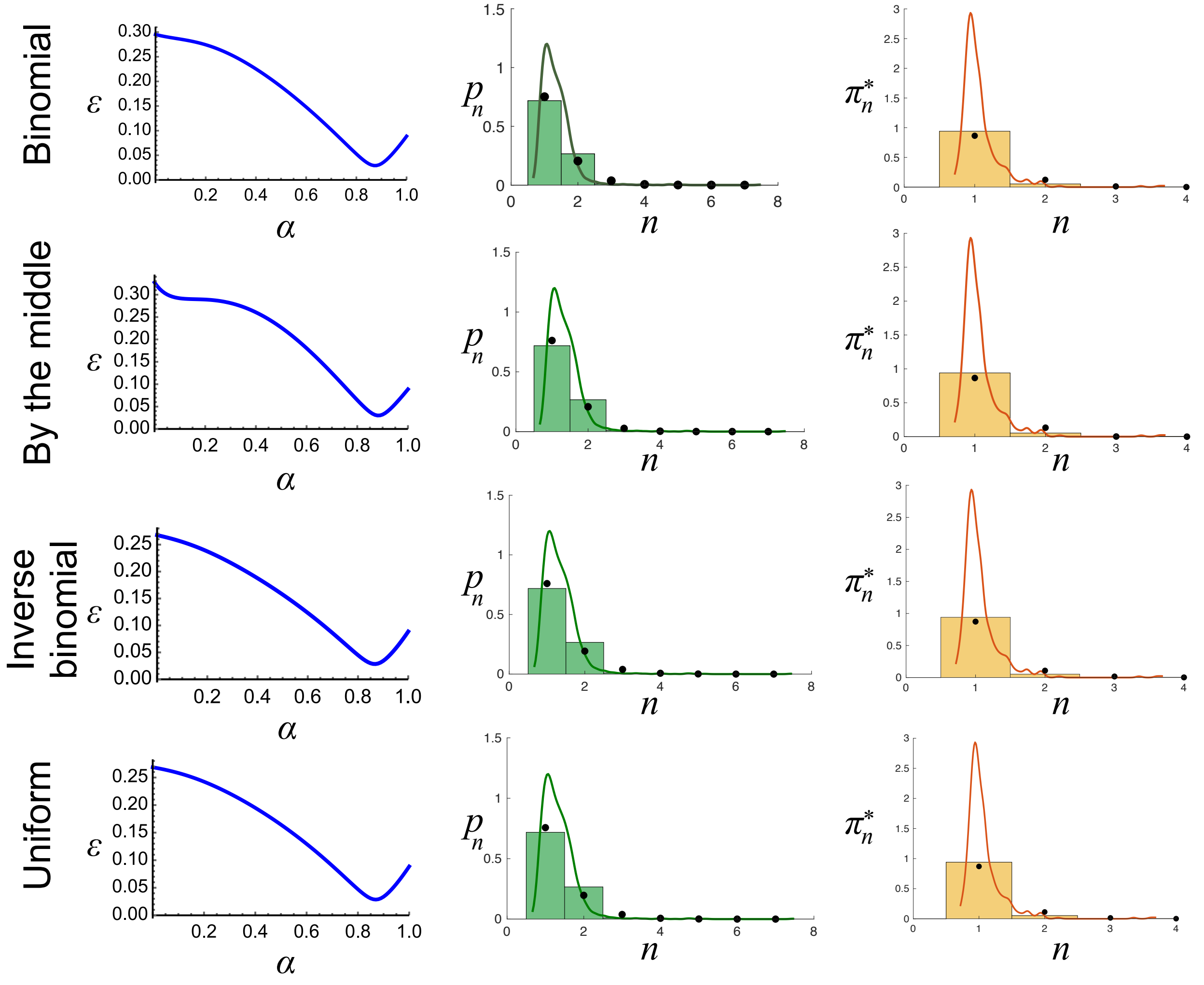

Figure S7. Fitting experimental data using different division patterns. The experimental cell size, p_n, and cell size at birth, π_n^*, probabilities (histograms) were simultaneously fitted to their theoretical counterparts (black dots) using an error, ϵ, minimization approach in order to find the value of the division efficiency α (see text). The binomial, inverse binomial, and uniform division patterns provided $\alpha = 0.87$ whereas the division by the middle pattern yielded $\alpha = 0.88$.

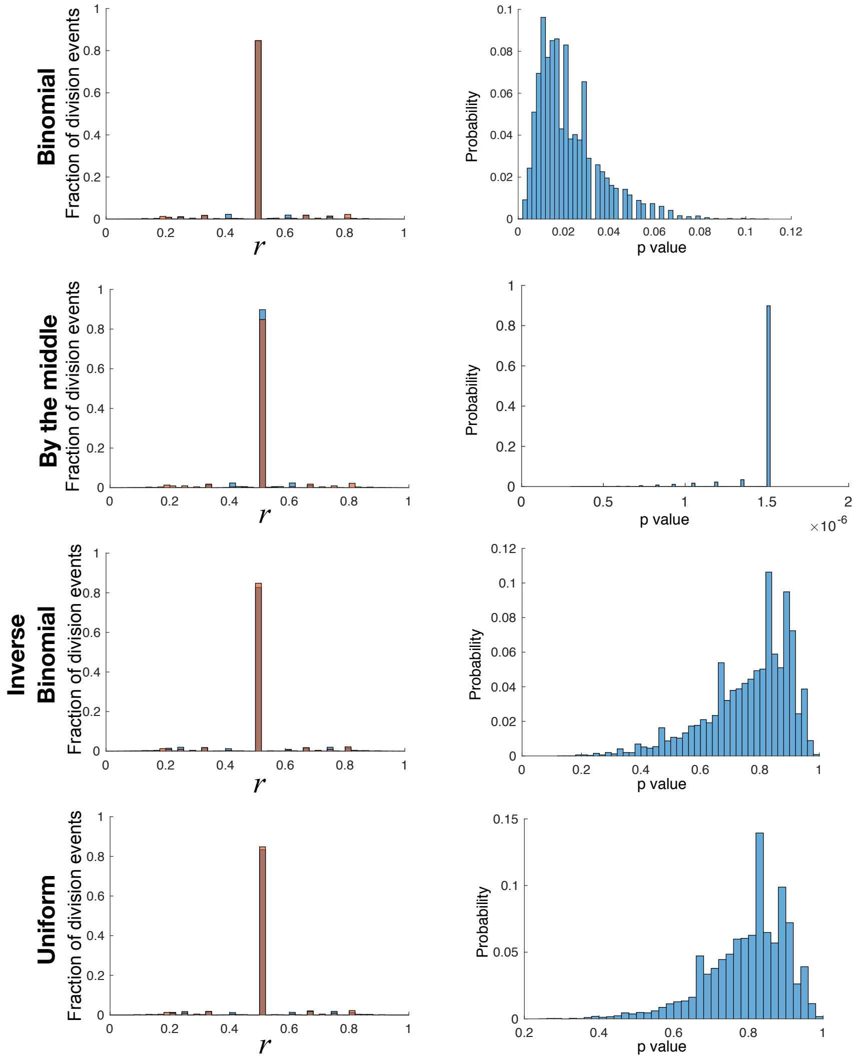

Figure S8. Characterization of the division ratio distribution by means of the Kolmogorov-Smirnov (KS) test, see Methods. We tested the 4 division patterns discussed in the main text. Left column: division ratio distributions. The orange histogram (repeated in all the plots) accounts for 4108 experimental division events (data from [2]). Each blue histogram accounts, depending on the division pattern, for the numerical data of a single stochastic sample. Right column: p-value histogram obtained from KS tests. Binomial, $\langle p \rangle = 0.02 \pm 0.01$. Division by the middle, $\langle p \rangle = (1.5 \pm 0.1) \cdot 10^{-6}$. Inverse binomial, $\langle p \rangle = 0.8 \pm 0.1$. Uniform, $\langle p \rangle = 0.8 \pm 0.1$. As for the percentages of rejection of the null hypothesis (identical distributions): Binomial, 96%. Division by the middle, 100%. Inverse binomial, 0%. Uniform, 0%.

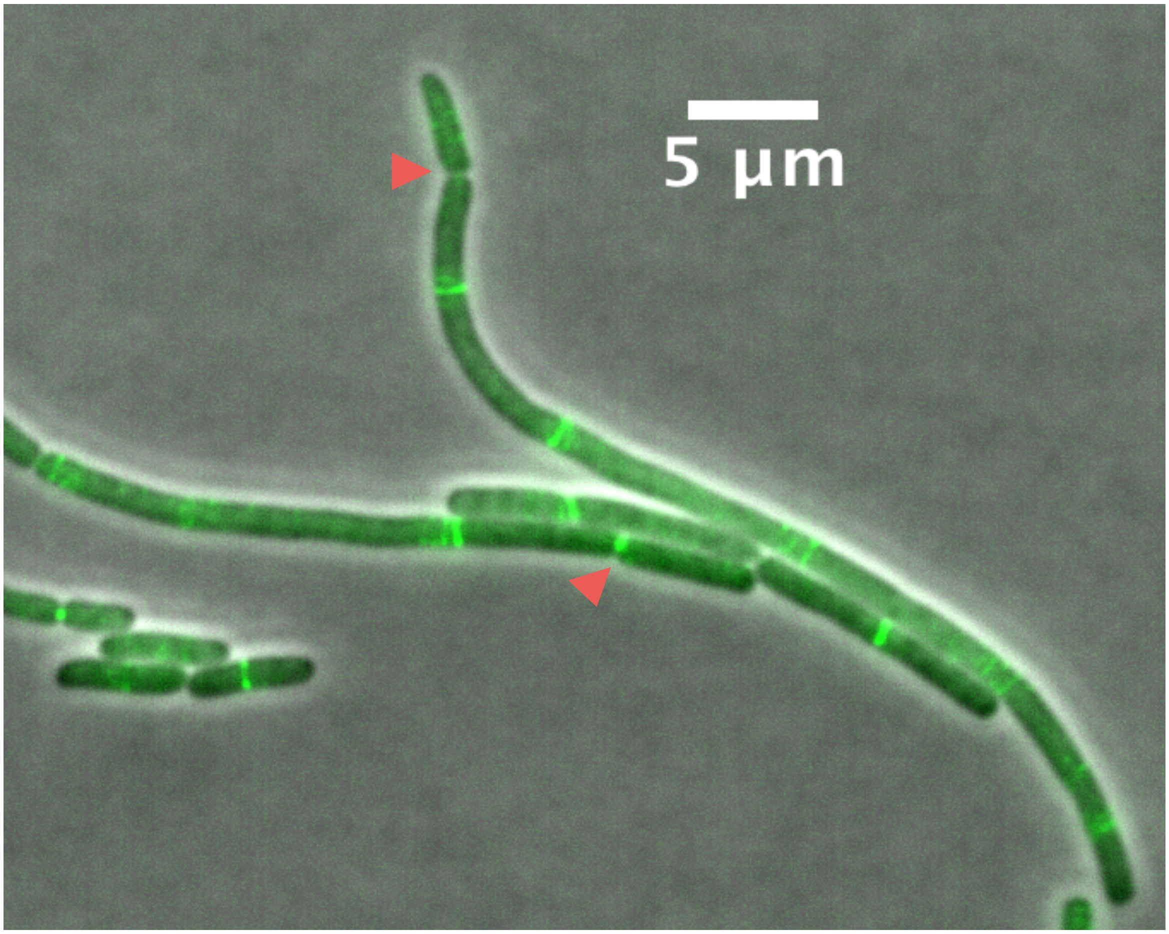

Figure S9. Filamentous cells (MGZ202) display clear asymmetric divisions and in most cases they divide close to the poles (red triangles). Fluorescent signal: FtsZ:GFP.

SUPPLEMENTARY MOVIES

Movie S1. *E.coli* MG1655 cells growing on a LB agarose pad (frame rate: 7 frames/sec.; time lapse interval: 2 mins.).

SIMULATION CODE AND RAW DATA

The FigShare Data repository http://doi.org/10.6084/m9.figshare.5827635 includes a Wolfram Mathematica code to simulate the trajectories of individual cells that follows the Markovian growth/division model using different division patterns and additional files containing the raw data for generating all figures but Fig. S8 (data from Tan's group, [2]).

[1] Walsh, J. C., Angstmann, C. N., Duggin, I. G. & Curmi, P. M. Molecular interactions of the min protein system reproduce spatiotemporal patterning in growing and dividing escherichia coli cells. *PloS one* **10**, e0128148 (2015).

[2] Wehrens, M. *et al.* Size laws and division ring dynamics in filamentous escherichia coli cells. *Current Biology* **28**, 972–979 (2018).
